# Synthesis of Fe_2_O_3_/γ-Al_2_O_3_ via Sol-Gel Method for Congo Red Adsorption: Kinetic Analysis and DFT Insights

**DOI:** 10.3390/nano16130814

**Published:** 2026-07-01

**Authors:** Yiwang Tang, Hongxia Wang, Junchao Zhang, Yuning Ma, Xiyao Tian, Xintong Liu, Xiulan Xin

**Affiliations:** Department of Daily Chemical Engineering, School of Light Industry Science and Engineering, Liangxiang Campus, Beijing Technology and Business University, Beijing 102488, China

**Keywords:** adsorbent, aluminum oxide, iron oxide composite, mesoporous material, monolayer adsorption

## Abstract

With the growing emphasis on environmental sustainability, the proper treatment of industrial wastewater and the protection of groundwater resources have become pressing global concerns. Congo red (CR), a widely used azo dye, enters water bodies via wastewater discharge, posing persistent ecological risks to surface and groundwater systems. Adsorption, as a direct and sustainable remediation approach, necessitates the development of high-performance adsorbents to inhibit CR migration into groundwater. In this study, a Fe_2_O_3_/γ-Al_2_O_3_ composite was synthesized via sol-gel method for efficient CR adsorption, thereby mitigating groundwater contamination risk. The composite exhibited a high specific surface area (246.22 m^2^/g) and a maximum adsorption capacity of 1027.72 mg/g. Adsorption behavior followed the pseudo-second-order kinetic and Langmuir isotherm models, consistent with chemisorption-driven monolayer adsorption. The Weber–Morris intraparticle diffusion model confirmed rapid initial surface adsorption, beneficial for practical groundwater remediation. pH-dependent adsorption efficiency further indicated the role of electrostatic interactions, informing process optimization under varying groundwater chemistries. DFT calculations demonstrated that Fe_2_O_3_/γ-Al_2_O_3_ possesses a higher adsorption affinity for CR than γ-Al_2_O_3_. Collectively, Fe_2_O_3_/γ-Al_2_O_3_ shows strong potential as a novel, efficient adsorbent for CR interception and groundwater quality protection.

## 1. Introduction

**Figure 1:**
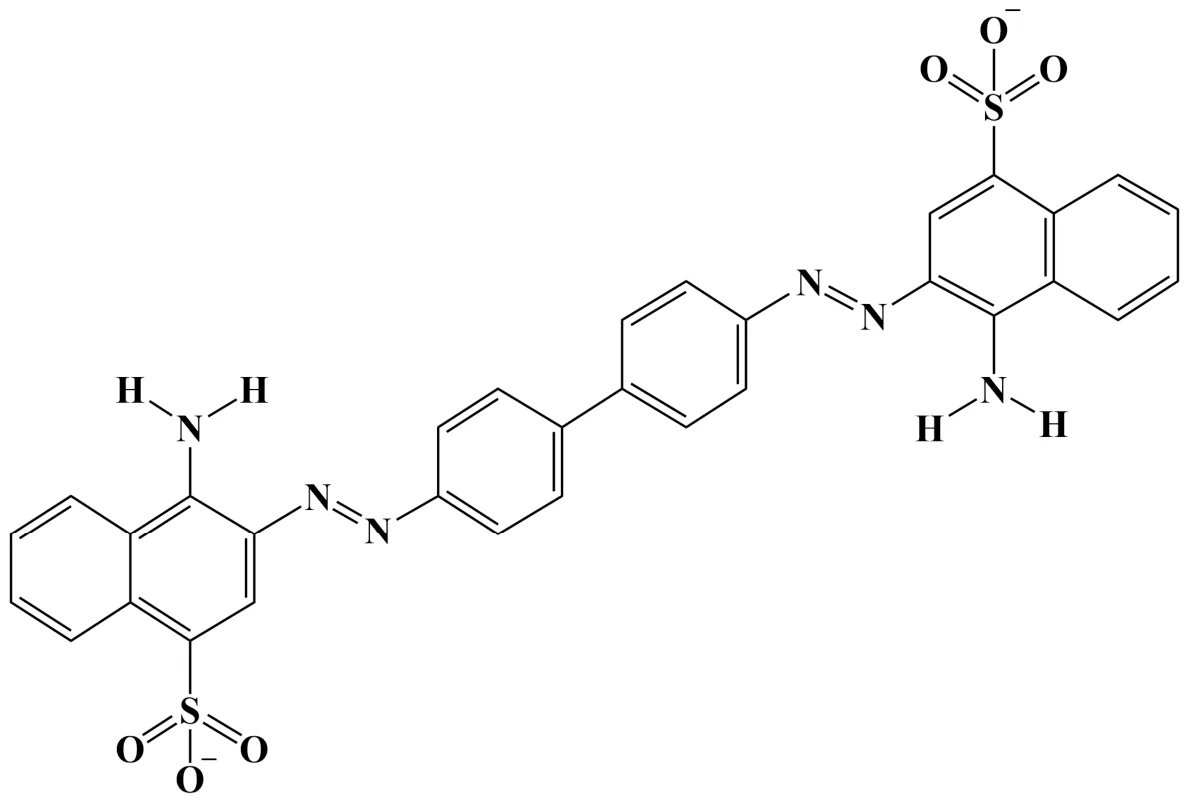
The structural formula of Congo red.

Given the importance of a hygienic living environment and access to safe drinking water for health and well-being, groundwater—as a key source for these needs and for industrial and agricultural activities—is fundamental to societal development. However, the escalating demand for water is expected to result in a corresponding rise in the quantity of wastewater produced, which poses a significant contamination risk to aquifers through seepage and runoff [1]. This interplay intensifies the pressure on this vital resource, making its protection a paramount concern. Wastewater treatment constitutes a critical component of industrial processes, as the presence of hazardous contaminants in wastewater adversely affects multiple environmental media, including the atmosphere, soil, and aquatic systems [2]. Azo dyes, particularly Congo red (CR), are widely used across various industrial sectors such as textile production, food processing, and leather manufacturing. Owing to its prevalence as a common anionic azo dye in these industries, substantial quantities of CR are released into the environment via industrial effluents [3]. Owing to the complex molecular architecture of CR, traditional biological, physical, and chemical treatment approaches for dye-containing effluents demonstrate limited effectiveness to varying extents [4].Traditional decontamination techniques, including ion exchange [5,6], biodegradation [7], and adsorption utilizing clays and activated carbon [8], have been established in previous research for the elimination of these detrimental substances. Adsorption is considered a highly effective technique for the elimination of dye contaminants from water bodies, owing to its superior efficiency, economic viability, operational simplicity, and the absence of secondary pollution [9]. Carbon [10], polymer [11], and metal (hydr) oxide materials [12] have been utilized for the removal of contaminants in carbon removal processes. The adsorption performance of various adsorbents for CR (Figure 1) is governed by multiple factors, including their morphology, structural characteristics, specific surface area, surface charge, and intrinsic adsorption affinity. Given that CR molecules carry a negative charge in aqueous solutions, adsorbents with a positively charged surface are particularly favorable for CR adsorption [13].

In recent years, the exploration of novel oxide-based systems has extended well beyond conventional single-metal oxides, driven by the pursuit of enhanced adsorptive and catalytic functionalities. For example, complex oxides such as wide-bandgap polycrystalline ferro-pseudobrookite (FeTi_2_O_5_) have been synthesized via sol-gel methods, with systematic investigations into annealing effects on their structural and optical properties [14]. Among conventional adsorbent materials, activated carbon exhibits considerable effectiveness in the removal of a wide range of organic and inorganic pollutants, including dyes and pigments. However, the utilization of this adsorbent in wastewater treatment is limited by its high cost and the difficulties encountered in its regeneration [15]. As a result, a wide range of alternative adsorbent materials have been explored for the purpose of removing dyes from aqueous solutions. These alternatives include activated carbon derived from low-cost carbon sources [16], fly ash [17], orange peel [18], waste red mud [19], and clay minerals [20], among others. Beyond traditional unmodified adsorbents, various modified adsorbents have been developed; however, they still present certain inherent limitations. For instance, metal–organic frameworks (MOFs) demonstrate particular constraints, including reduced surface area and restricted pore dimensions, which limit their adsorption capacity for macromolecular dyes. Additionally, the inherent structural defects within MOFs negatively impact their hydrophobic characteristics [21]. Furthermore, certain metal oxides exhibit effective adsorption properties for CR, including nanoparticles such as Fe_2_O_3_ [22], Al_2_O_3_ [23], Cu_2_O [24], and MgO [25]. Nevertheless, these materials typically exhibit restricted pore volume and pore size, along with comparatively low specific surface area (S_BET_) and maximum adsorption capacity (*Q_m_*). At present, γ-Al_2_O_3_-based materials demonstrate considerable potential in adsorption applications, attributable to their low cost and straightforward synthesis procedures [26,27]. Despite this, *Q_m_* stability, and S_BET_ of unmodified γ-Al_2_O_3_ remain below anticipated standards. Therefore, it is imperative to either modify γ-Al_2_O_3_ or enhance the synthesis methodology to improve its stability, S_BET_, and *Q_m_*.

To address the aforementioned gaps, we report the synthesis of mesoporous Fe_2_O_3_/γ-Al_2_O_3_ nanocomposites via sol-gel and one-pot methods using aluminum n-hexoxide and ferric chloride hexahydrate as precursors for CR adsorption. The novelty of this work is threefold:

(1) Inorganic iron source. Unlike conventional routes relying on organic iron precursors, we employ inexpensive ferric chloride hexahydrate, which simplifies the synthesis, reduces costs, and minimizes organic solvent pollution-a practical strategy scarcely explored in the literature.

(2) Synergistic parameter optimization. While previous studies typically vary a single factor, we systematically investigate the combined effects of alcohol-to-water ratio, aging time, and aging temperature on the textural properties of the composite-a parameter combination not previously reported for Fe_2_O_3_/γ-Al_2_O_3_ systems.

(3) Superior performance with mechanistic insights. The optimized composite achieves a maximum CR adsorption capacity of 1027.72 mg/g, substantially exceeding pristine γ-Al_2_O_3_ and Fe_2_O_3_. Beyond this performance, we provide a comprehensive analysis of the kinetics, isotherm, and underlying adsorption mechanisms, offering a systematic framework for anionic dye removal on mixed-metal-oxide adsorbents.

Collectively, these contributions distinguish our work from existing literature and provide both fundamental insights and practical guidance for designing cost-effective, environmentally benign adsorbents for wastewater treatment.

The remainder of this paper is organized as follows. Section 2 details the experimental procedures, including the synthesis of the Fe_2_O_3_/γ-Al_2_O_3_ nanocomposites, the characterization techniques employed, and the batch adsorption experiments conducted for CR removal. Section 3 presents the results of material characterization and adsorption performance, along with DFT calculations to reveal the molecular-level mechanisms. Section 4 provides a comprehensive discussion of the findings, integrating experimental and computational results. Finally, Section 5 summarizes the key conclusions of this work and provides an outlook on future research directions.

## 2. Materials and Methods

All reagents utilized in this study are of analytical grade purity and have not been subjected to any purification processes prior to their application. n-hexanal (purty: 99% from Shanghai MacLean Biochemical Technology Co., Ltd.; Shanghai, China), isopropyl alcohol (purty of 99.5% from Shanghai MacLean Biochemical Technology Co., Ltd.), Aluminum isopropanol (Shanghai MacLean Biochemical Co., Ltd.), Aluminum n-hexanol (Shandong Yunneng Catalysis Technology Co., Ltd.; Dongying, China), FeCl_3_ 6H_2_O (Sinopharm Chemical Reagent Co., Ltd., Delaware, USA), Iron (Ⅲ) Ethoxide (purity: 99.5% from Shanghai Titan Scientific Co., Ltd.; Shanghai, China), Congo Red (Shanghai Yuanye Biotechnology Co., Ltd.; Shanghai, China). Deionized water and ultrapure water, self-made in the laboratory. And all the water used in this experiment was produced by ZYpureEDIA-100-UP system.

X-ray diffractometer (XRD, TD-3500 type), Dandong Tongda Technology Co., Ltd., Dandong, China; Fourier Transform Infrared spectroscopy (FT-IR, NA 370 type), Thermofisher Company, Delaware, USA; X-ray photoelectron spectroscopy (XPS, Kratos Axis Supra TM), Shimadzu, Kyoto, Japan; Thermogravimetric and simultaneous thermal analyzer (TGA, TGA/DSC^3+^ type), Mettler Toledo Corporation, Columbus, USA; specific surface area volume and pore size analyzer (ASAP 2460 model), Micromeritics Corporation, Norcross, USA; Ultraviolet-visible spectrophotometer (UV-3600 type), Shimadzu Corporation; Scanning Electron Microscope (SEM, Nova NanoSEM450), FEI, Hillsboro, USA; Transmission Electron Microscope (TEM, JEM-2100 Plus), JEOL, Kyoto, Japan; Vibrating Sample Magnetometer (VSM, Lakeshore 8404), Lakeshore, Columbus, USA; Inductively Coupled Plasma Optical Emission Spectrometry, (ICP-OES, Agilent 7250), Agilent, Santa Clara, USA; pH meter (PHS-3C), LeiCi, Shanghai, China; Zeta Potential Analyzer, (ZEN3600), Malvern, Malvern, UK; Electron Paramagnaetic Resonance (EPR, EMXplus-9.5/12 5000), Bruker, Karlsruhe, Germany.

It is worth noting that the synthesis strategy employed in this work may offer certain practical advantages over conventional preparation methods. For instance, the use of ferric chloride hexahydrate as an iron source provides a readily available and cost-effective alternative to organic iron precursors, which are often expensive and moisture-sensitive. In addition, the direct incorporation of the iron precursor during the hydrolysis step allows the composite to be obtained in a single-pot operation, which may simplify the synthesis procedure relative to multi-step impregnation routes. Furthermore, we attempted to systematically examine the combined effects of the alcohol-to-water ratio, aging time, and aging temperature on the textural properties of the composite; to the best of our knowledge, such a comprehensive investigation has not been previously reported for the Fe_2_O_3_/Al_2_O_3_ system.

Preparation of γ-Al_2_O_3_: The γ-Al_2_O_3_ support (labeled as AO, with an alcohol-to-water molar ratio of 1:6) was prepared using a hydrolysis–azeotropic distillation technique. In a typical procedure, 33.0 g of aluminum n-hexoxide was thoroughly mixed in a 200 mL beaker with a heating jacket, and then transferred to a 200 mL four-neck round-bottom flask equipped with a mechanical stirrer, reflux condenser, and oil bath. While stirring continuously, 10.2 g of n-hexanol was added to the flask, and the oil bath was heated to 90 °C. Deionized water (43.2 g), preheated to 90–95 °C, was slowly added dropwise over 10–15 min. The hydrolysis reaction was maintained at 9 °C for 2 h under stirring, followed by an aging process at 95 °C for 12 h.

Next, 50 g of deionized water preheated to 95 °C was added to the mixture, and the temperature was increased to 135 °C to start azeotropic distillation, which continued for 2 h to remove the alcohol–water azeotrope. The solid product obtained was dried at 135 °C for 6 h, resulting in pseudo-boehmite as a white powder. Finally, the precursor was calcined in a muffle furnace at 550 °C using a controlled heating schedule: a 3 h ramp from room temperature to 550 °C, a 3 h hold at 550 °C, and a 3-h natural cooling to 25 °C, producing the final γ-Al_2_O_3_ material.

**Figure 2:**
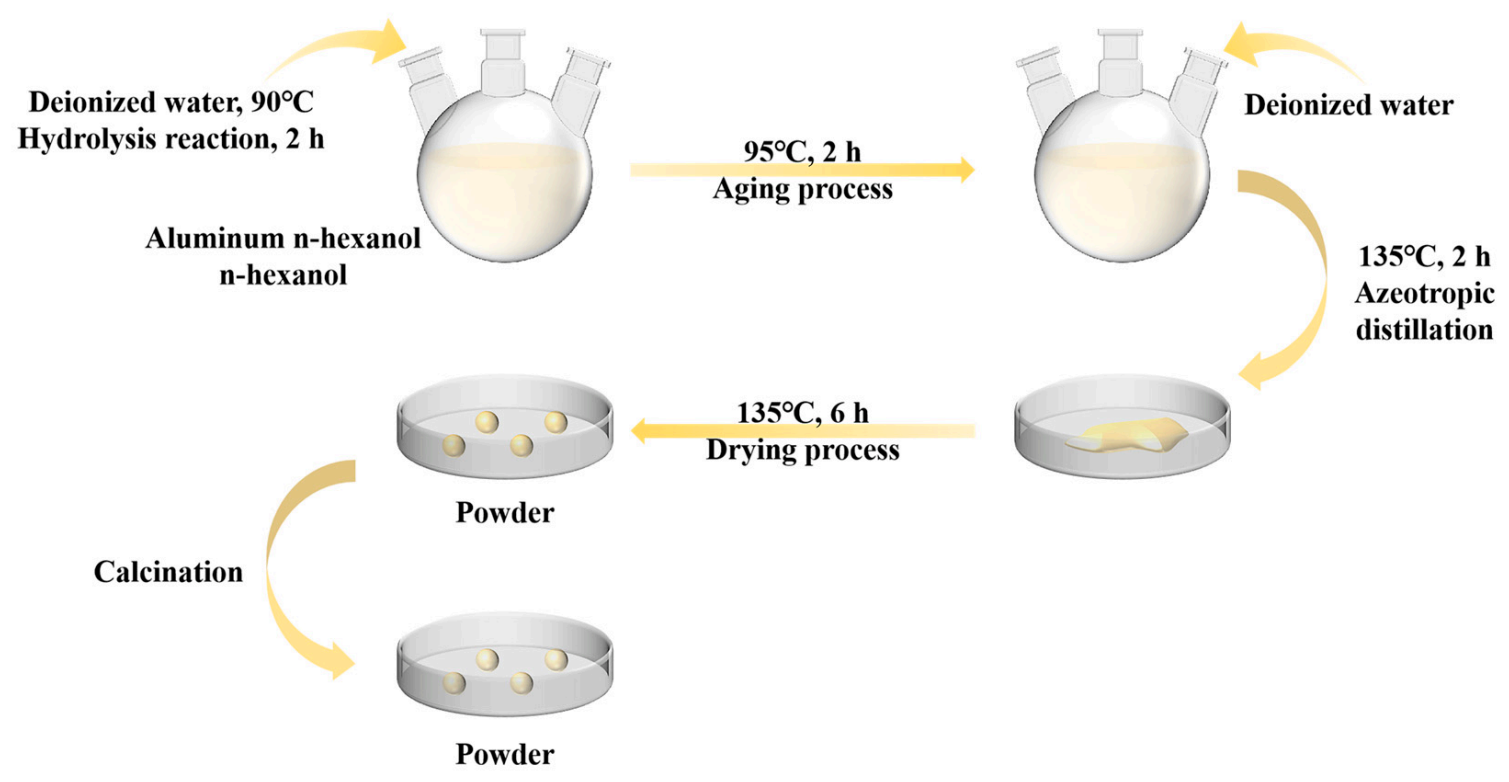
The process flow chart for synthesizing FA1100.

Preparation of Fe_2_O_3_/γ-Al_2_O_3_ (taking FA1100 as an example): A mixture of aluminum n-hexanol (33.0 g), FeCl_3_·6H_2_O (270 mg), and aluminum n-hexanol (10.2 g) was transferred to a four-necked round-bottom flask and immersed in a constant-temperature oil bath. Subsequent hydrolysis, aging, and calcination followed the same conditions as for pure γ-Al_2_O_3_ (Figure 2). The resulting Fe-Al oxide composite was designated FA1100, where F = Fe_2_O_3_, A = γ-Al_2_O_3_, and 1100 denotes an Fe/Al molar ratio of 1:100. The synthesis was carried out with a nominal Fe/Al molar ratio of 1:100 (based on precursor amounts).

This study investigated the effects of different aluminum and iron sources on the physicochemical properties and adsorption performance of Fe_2_O_3_/γ-Al_2_O_3_ composites. Using a fixed Fe/Al molar ratio of 1:100, mesoporous composites were synthesized with two aluminum sources (aluminum n-hexanol and aluminum isopropanol) and two iron sources (ferric chloride hexahydrate and ferric ethoxide). The composite FA1100 was prepared with aluminum n-hexanol and ferric chloride hexahydrate; FA1100-2 with aluminum n-hexanol and ferric ethoxide; and FA1100-1 with aluminum isopropanol and ferric chloride.

The XRD study of the as-prepared mesoporous material was carried out by employing a TD-3500 type at a scan rate (2θ) of 0.1°/s. The applied voltage was 10 kV and the current was 2 mA. Utilize XPS to analyze the concentrations of Fe, Al, and O within the sample. Utilize TGA to ascertain the temperature at which maximum weight loss, as well as the temperature associated with the transformation of crystal forms. The nitrogen adsorption technique was implemented to estimate S_BET_ using specific surface area volume and pore size analyzer. Before the nitrogen adsorption process, all samples were degassed at 300 °C for 6 h. The pore size distribution was analyzed using the cylindrical pore model. FT-IR spectroscopy examination was performed using instrument. FA1100 and AO captured SEM images at acceleration voltages of 10 kV and 15 kV respectively. The analysis of TEM was carried out under the condition of 20 kV. The elemental compositions of Fe and Al were analyzed using ICP-OES method, specifically employing the FA1100 test protocol. The magnetic force intensity of FA1100 was analyzed using VSM. The presence of oxygen vacancies during the synthesis process was analyzed using EPR spectroscopy. The potential measurement was conducted by preparing a suspension through the combination of AO and FA1100 with water, followed by analysis using a Zeta potential analyzer.

The adsorption kinetics of CR were investigated by dispersing 50 mg of adsorbent in 50 mL of CR solution (1000 mg/L) in a 50 mL conical flask under constant stirring at 25 °C, without pH adjustment. Aliquots (1 mL) were collected at predetermined intervals (0–72 h). After centrifugation, the supernatant was analyzed using a UV-3600 spectrophotometer at 497 nm following the Lambert–Beer law. The adsorption capacity and removal rate (*R_e_*, %) at each time interval (Table 1) were calculated using Formula 2.

Under the same conditions as the kinetic study (adsorbent dosage: 50 mg/50 mL), equilibrium adsorption of CR was investigated at initial concentrations ranging from 1000 to 2000 mg/L. After 72 h, residual CR concentrations were measured via UV-Vis spectrophotometry. Isotherm data were fitted to Langmuir, Freundlich, and Tempkin models. The effect of adsorbent dosage (10–50 mg) on CR adsorption was examined while keeping other parameters constant. For regeneration studies, 50 mg of adsorbent was added to 50 mL of CR solution (1000 mg/L) and stirred for 72 h. The mixture was centrifuged at 8000 rpm for 10 min, and the recovered solid was calcined following the established protocol to restore adsorption capacity. This procedure was repeated twice, each cycle using fresh CR solution (1000 mg/L) under equilibrium conditions. Supernatant CR concentrations were measured after each regeneration cycle.

## 3. Results

### 3.1. Morphology and Structure Characterizations

**Figure 3:**
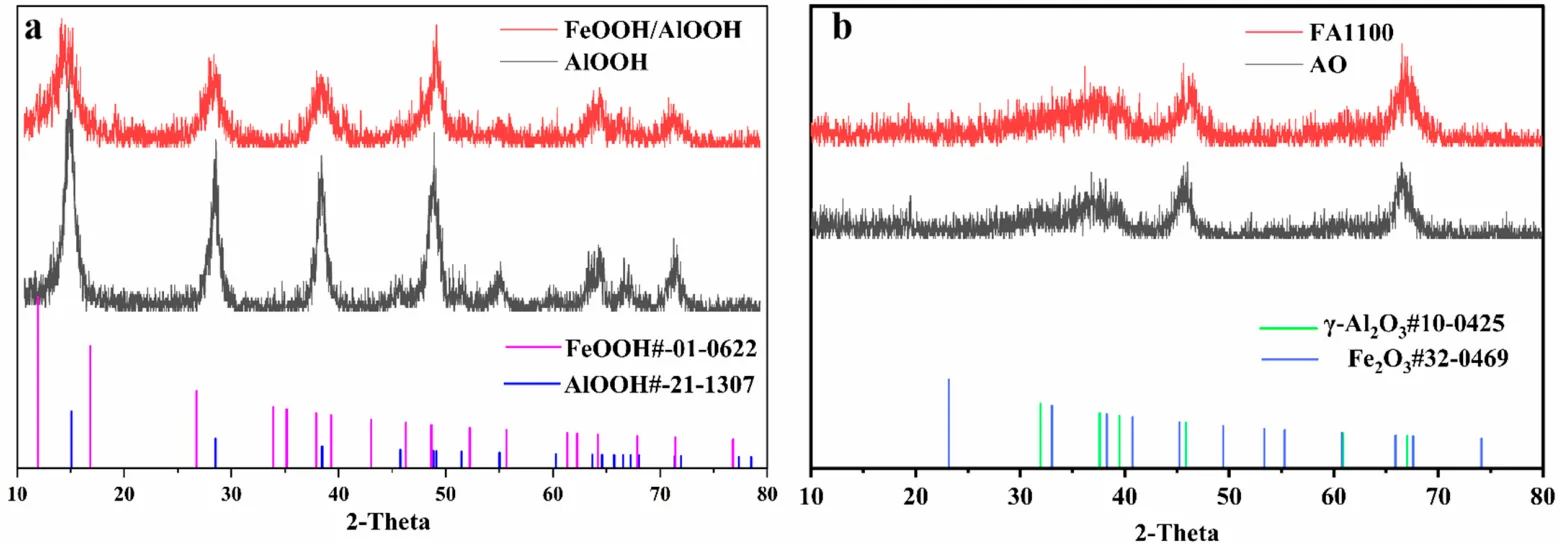
XRD patterns of (**a**) the precursor and (**b**) the final Fe_2_O_3_/γ-Al_2_O_3_ nanocomposite after calcination. The diffraction peaks are indexed according to the standard JCPDS cards: FeOOH (JCPDS No. 01-0622), AlOOH (JCPDS No. 21-1307), γ-Al_2_O_3_ (JCPDS No. 10-0425), and Fe_2_O_3_ (JCPDS No. 32-0469).

The adsorbent was synthesized via a one-step procedure (Figure 2). FeOOH/AlOOH was first prepared using the sol-gel method, and then calcined at 550 °C in a muffle furnace. The samples were characterized by XRD (Figure 3). Comparison of peak positions with the PDF database confirmed that the precursor consisted of FeOOH and AlOOH. After calcination, the diffraction peaks corresponding to AlOOH disappeared and were replaced by those of γ-Al_2_O_3_, indicating complete transformation of the alumina precursor. However, no distinct crystalline Fe_2_O_3_ peaks were observed in the calcined sample. The characteristic peaks of γ-Al_2_O_3_ are sharp and well-defined, indicating high crystallinity and purity. After Fe incorporation, the γ-Al_2_O_3_ peaks exhibited slight broadening and decreased intensity, while no additional peaks corresponding to Fe_2_O_3_ or FeAlO_3_ spinel phase were detected. The absence of distinct Fe_2_O_3_ diffraction peaks suggests that the Fe species may be highly dispersed on the γ-Al_2_O_3_ surface, likely present as amorphous FeOₓ, isolated Fe(III) ions, or Fe–O–Al surface species, rather than as well-crystalline bulk Fe_2_O_3_ particles. However, we acknowledge that further characterization (e.g., EXAFS or Mössbauer spectroscopy) would be required for a more definitive assignment of the Fe species.

**Figure 4:**
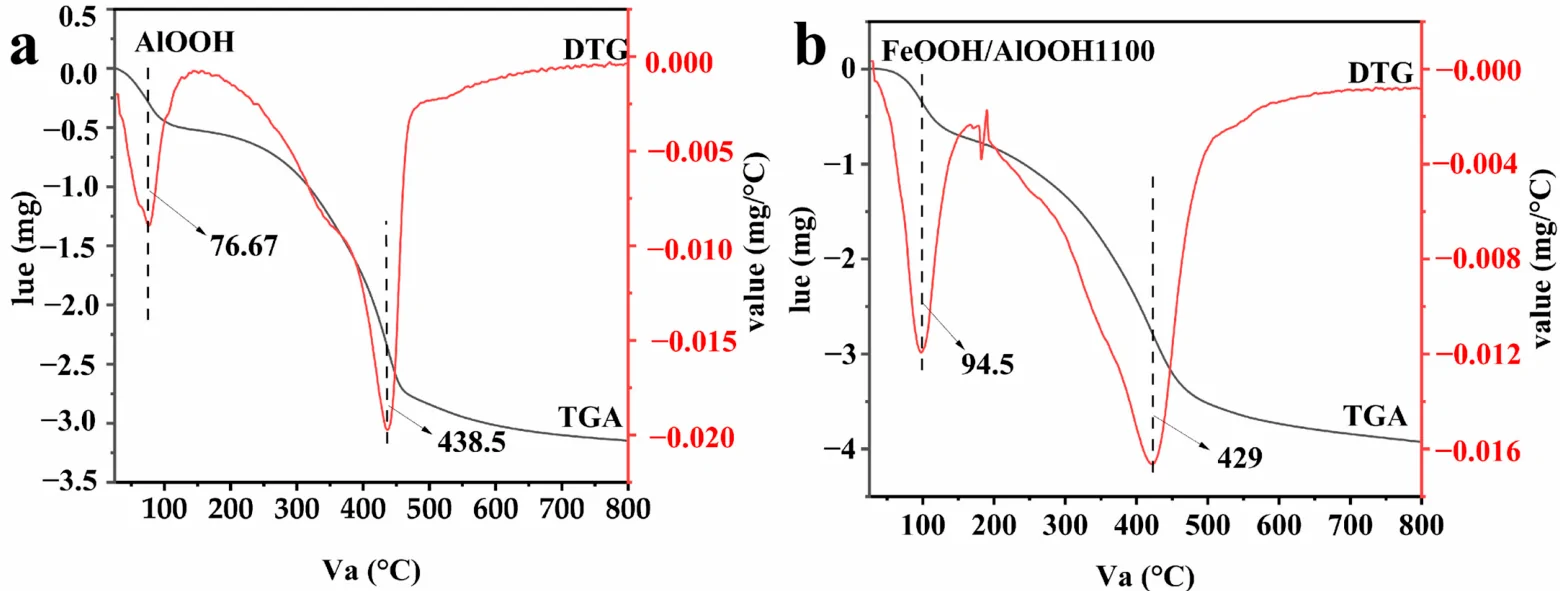
TGA and DTG analysis of (**a**) AlOOH and (**b**) FeOOH/AlOOH1100.

To precisely determine the crystal phase transition temperature of Fe_2_O_3_/γ-Al_2_O_3_, TGA was performed to accurately verify the crystal phases of γ-Al_2_O_3_ and Fe_2_O_3_/γ-Al_2_O_3_. These phases originated from AlOOH and FeOOH/AlOOH, corresponding to AO and FA1100, respectively (see Figure 4). The FeOOH/AlOOH1100 sample exhibits a maximum endothermic peak at 94.5 °C, which is ascribed to the desorption of surface-adsorbed water. In contrast, the FeOOH/AlOOH sample demonstrates its most prominent endothermic peak at 429 °C, corresponding to the dehydroxylation process and the subsequent transformation to the γ phase. γ-Al_2_O_3_ was chosen as the raw material for the adsorbent due to its high specific surface area, substantial porosity, and other advantageous characteristics [28]. Conversely, AlOOH exhibits an endothermic reaction peak at 438.5 °C, indicative of its transformation into γ-Al_2_O_3_. This variation can be explained by considering the dehydroxylation process of FeOOH, which occurs at a comparatively lower temperature of 225 °C [29]. This observation further explains why the incorporation of a small quantity of FeOOH into AlOOH results in a shift in the maximum endothermic peak to a lower temperature. Additionally, the consistent XRD and TGA results confirm the successful synthesis of Fe_2_O_3_/γ-Al_2_O_3_.

**Figure 5:**
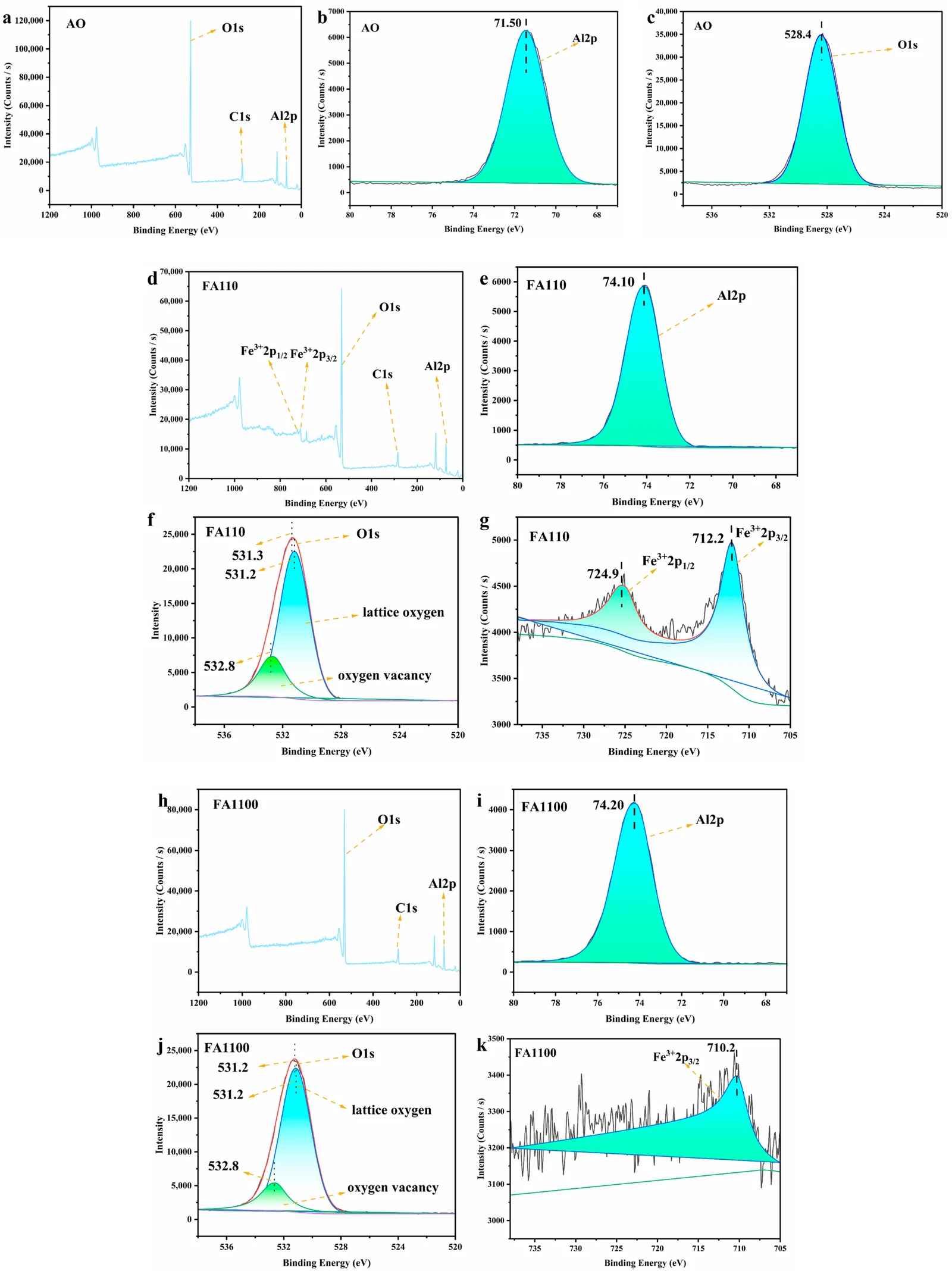
XPS image of AO (**a**–**c**), FA110 (**d**–**g**) and FA1100 (**h**–**k**).

Furthermore, XPS is an important means of characterizing the chemical state of material surfaces. In this study, to the extremely low concentration of the Fe element in FA1100, the XPS signal intensity is relatively weak. Therefore, the XPS spectrum of FA110 was selected to elucidate the bonding configuration of the Fe element within the complex. As illustrated in Figure 5, the measured energies for these orbitals are as follows: Fe^3+^ (2p_3/2_) at 712.05 eV, Fe^3+^ (2p_1/2_) at 725.34 eV, O (1s) at 531.51 eV, and Al (2p) at 74.31 eV [30]. Among the three elements Fe, Al, and O, the sawtooth pattern observed in the XPS energy spectrum peak of Fe may also suggest a low concentration of the doped Fe element. Figure 5g illustrates that the iron present in the synthesized composite material exists predominantly in the Fe(III) oxidation state. This observation aligns with previously reported findings in the literature, which indicate that the XPS spectra of iron oxide compounds calcined in air at temperatures exceeding 550 °C primarily exhibit Fe^3+^ species [31]. Upon the addition of Fe_2_O_3_ and examination of Figure 5b,e,i, it is evident that the binding energy of the Al (2p) orbital shifts from 71.50 eV to 74.20 eV. This shift suggests the presence of interactions beyond the conventional Al–O–Al bonding that influence the Al (2p) binding energy. Furthermore, analysis of Figure 5c,f,j reveals an increase in the binding energy of the O (1s) orbital. The emergence of double peaks in Figure 5f,j implies the possible formation of oxygen vacancy, which contributes to the enhancement of the O (1s) orbital binding energy. Typically, the surface oxygen species present in oxygen carriers are categorized into two distinct types: lattice oxygen and surface-adsorbed oxygen or oxygen vacancy [32]. The presence of oxygen vacancies suggests possible asymmetric Fe-O-Al bond formation during synthesis. Due to the electronegativity difference between Al and Fe, the electron cloud density around Al increases, enhancing the bond energy associated with the Al (2p) orbital. Quantitative analysis (Table 2) shows the contents of Fe^3+^ (2p_3/2_), O (1s), and Al (2p) as 0.69%, 12.33%, and 7.56%, respectively, yielding an Fe/Al molar ratio of approximately 1:10, consistent with the theoretical value.

**Figure 6:**
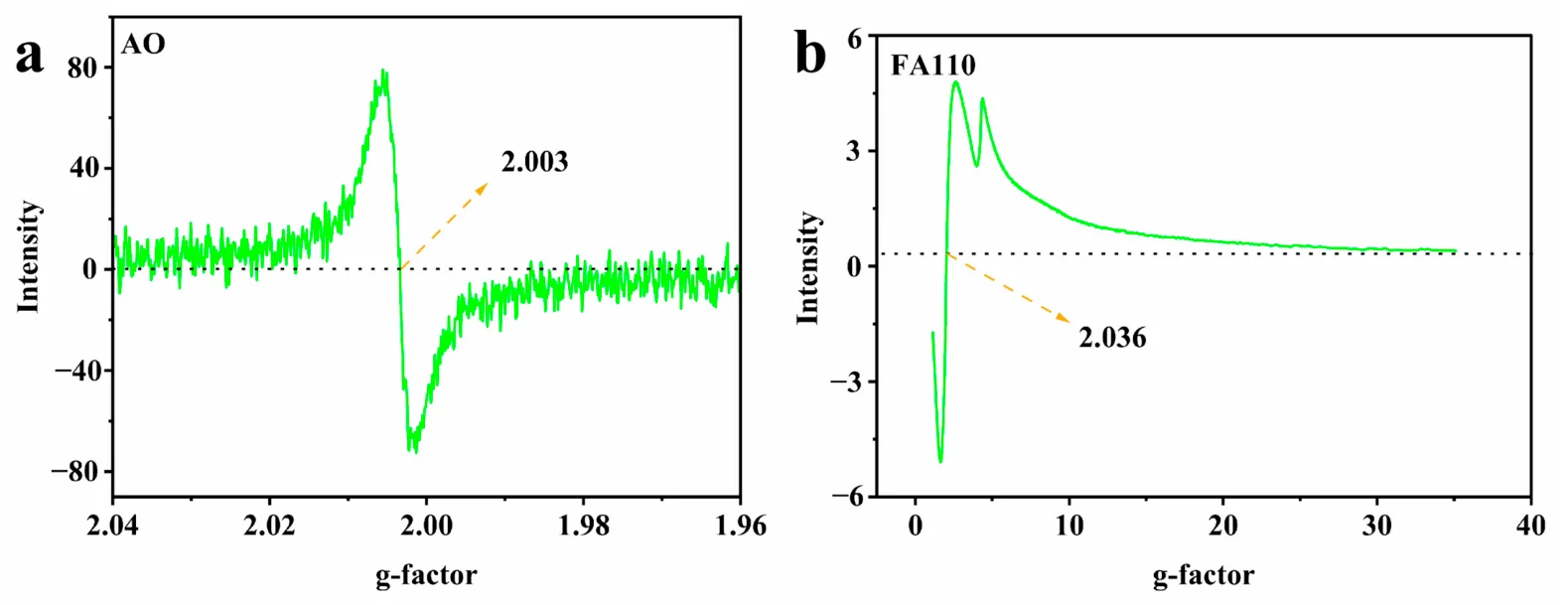
EPR image of (**a**) AO and (**b**) FA 110.

To further interpret the bond energy shifts in Al (2p) and O (1s) observed by XPS, EPR analysis was conducted to detect oxygen vacancies generated during the synthesis of FA110 and AO. Oxygen vacancies were present throughout the process. The g value of AO (Figure 6a) is 2.003, close to that of free charge (2.0023), indicating minimal vacancy generation by Al atoms and retention of three-coordinate oxygen, with dominant Al-O-Al bonding and negligible lone-pair displacement. In contrast, the g value of FA110 (Figure 6b) is 2.036, suggesting that Fe atoms alter the electronic environment. Due to Fe’s lower electronegativity relative to Al, oxygen electrons are drawn toward Al sites, increasing oxygen vacancy concentration. The shoulder peak on the right side of the vacancy signal arises from the intrinsic paramagnetism of Fe atoms, whose spectral feature overlaps with that of oxygen vacancies. These findings are generally consistent with the XPS observations and suggest a possibility that Fe species may be incorporated into the alumina framework, potentially introducing crystal defects and Fe–O–Al interactions. Nevertheless, we acknowledge that such interpretations based solely on these results remain speculative, and further structural characterization would be needed for a more definitive conclusion.

**Figure 7:**
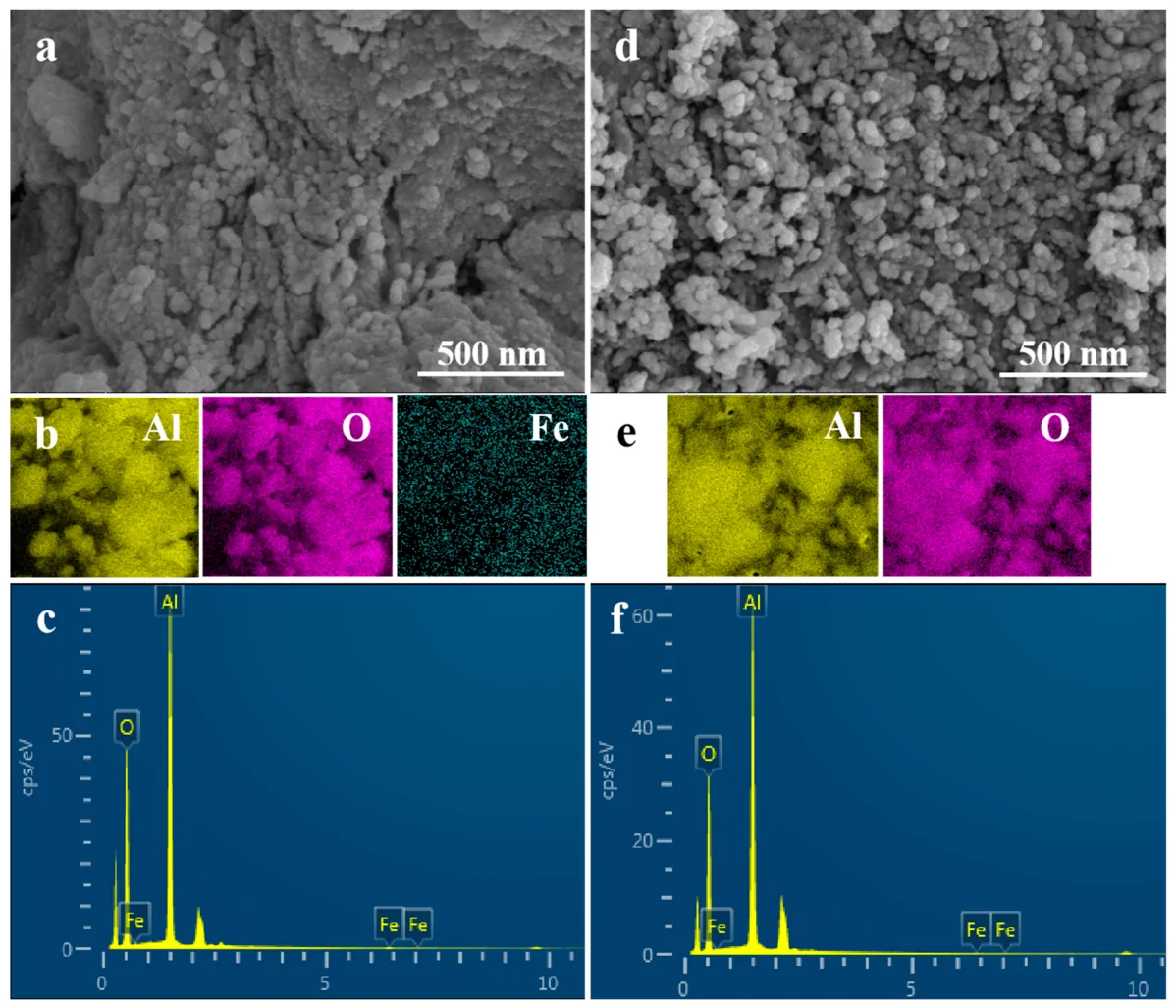
SEM image of (**a**) FA1100 and (**d**) AO, EDS mapping of (**b**) FA1100 and (**e**) AO, EDS spectra of (**c**) FA1100 and (**f**) AO.

Morphology and surface element distribution were obtained by SEM and EDS analyses, which were performed on AO and FA1100. The findings of this research are presented in Figure 7 and Table 3. Figure 7a,d illustrate that the synthesized FA1100 and AO are present in the form of nanospheres. The spherical morphology likely promotes increased interaction between FA1100 and the dye molecule CR, thereby improving the adsorption efficiency. Figure 7b depicts the distribution and concentration of Fe, Al, and O elements within FA1100, revealing a uniform distribution of iron throughout the sample. Furthermore, Table 3 provides the measured concentrations of iron and aluminum, recorded at 0.43% and 36.84%, respectively, which closely align with the theoretical molar ratio of 1:100. These observations are also corroborated by the results obtained from XPS analysis.

**Figure 8:**
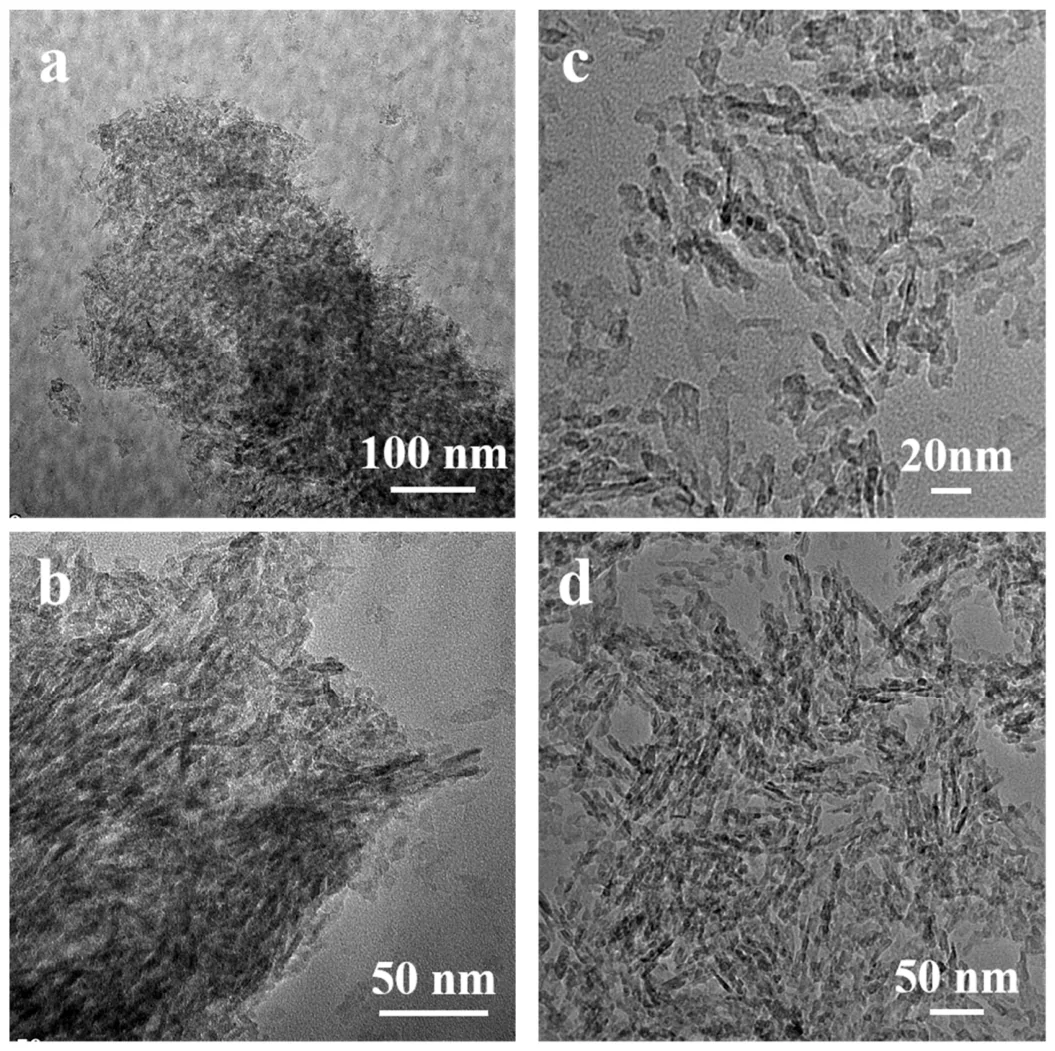
TEM image of (**a**,**b**) of FA1100, (**c**,**d**) of AO.

To enhance the understanding of the morphological properties of AO and FA1100, TEM analysis was performed. As illustrated in Figure 8, both AO and FA1100 exhibit nanorod structures. This observation contrasts with the results obtained from SEM, which may be attributed to the aggregation of multiple rod-shaped molecules of AO and FA1100, resulting in the formation of microspheres at the 500 nm scale observed in the SEM images. The atomic concentrations of Fe and Al in FA1100 were quantified using ICP-OES, with the relevant parameters detailed in Table 4. The analysis revealed an Fe concentration of 1.63 ppm (measured at 240.49 nm) and an Al concentration of 66.23 ppm (measured at 308.22 nm), corresponding to an actual Fe/Al molar ratio of approximately 1:84. This value is slightly lower than the nominal synthesis ratio of 1:100 (based on precursor amounts), which may be attributed to the partial loss of Fe species during the multi-step synthesis process (including hydrolysis, aging, washing, and calcination). The measured composition is further supported by EDS analysis (approximately 1:86), while XPS results are consistent with the presence of both Fe and Al species in the composite.

**Figure 9:**
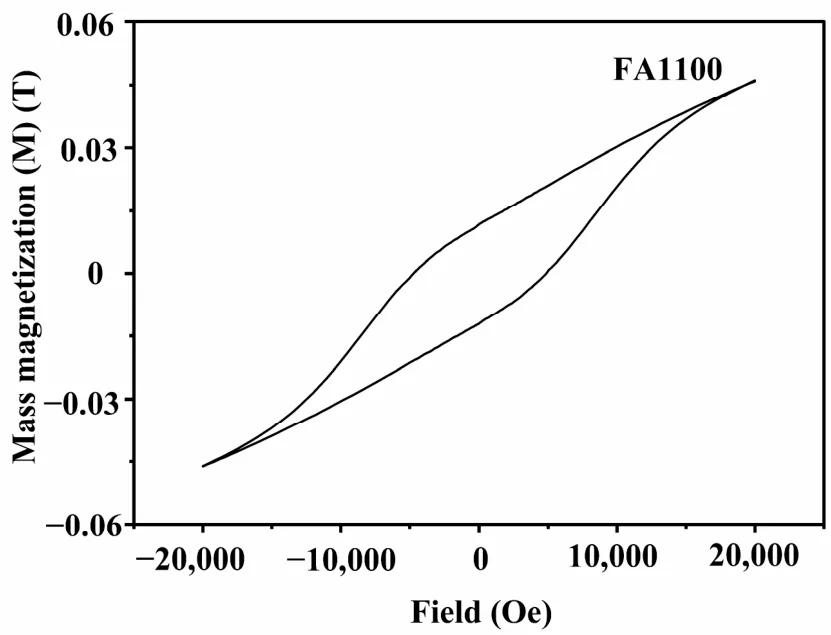
VSM image of FA1100.

Previous studies have demonstrated that magnetic adsorbents are effective in the adsorption and removal of organic pollutants and metal ions, owing to their rapid mass transfer rates and high adsorption capacities. Furthermore, enhancements in their adsorption performance have been reported [33]. To investigate the magnetic properties of FA1100, VSM measurements were performed, with the corresponding results presented in Figure 9. The data indicate that the magnetization curve of FA1100 ranges from −0.05T to 0.05T, thereby demonstrating that FA1100 possesses a degree of magnetic properties.

**Figure 10:**
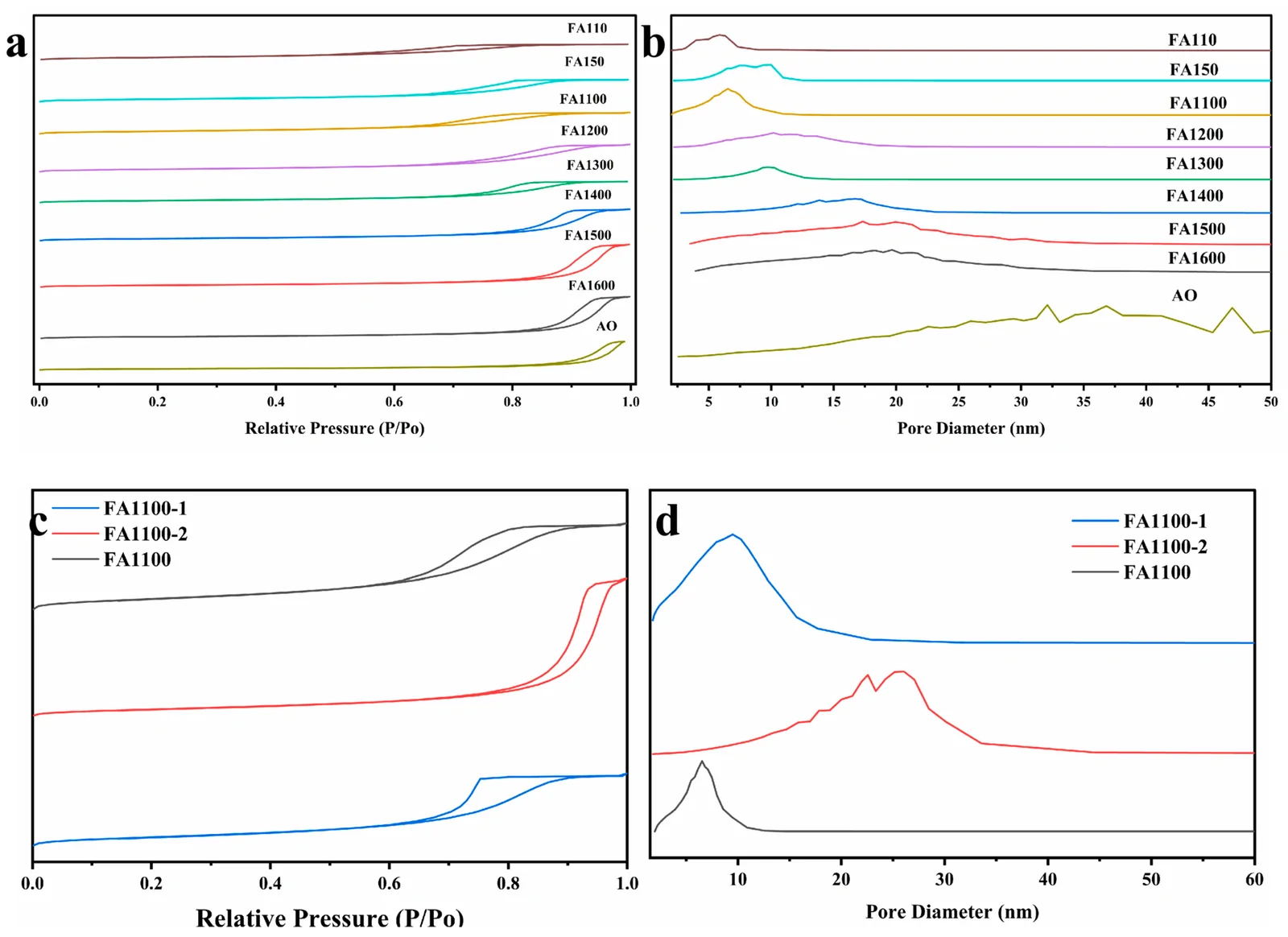
N_2_ adsorption-desorption curve of (**a**) different molar ratios of iron to aluminum and (**b**) different molar ratios of iron to aluminum and Pore Size stepwise curve of (**c**) different molar ratios of iron to aluminum and (**d**) different molar ratios of iron to aluminum. The data are presented as *y*-axis offset plots, where the vertical axis serves only as a relative scale for visual comparison, with no absolute physical meaning.

As discussed above, pore volume, pore size, and specific surface area are key factors that affects the adsorption efficiency. Consequently, pore volume, pore size and S_BET_ of the synthesized Fe_2_O_3_/γ-Al_2_O_3_ were measured, with the results illustrated in Figure 10. The BET analysis depicted in Figure 10a reveals the hysteresis loops corresponding to materials with varying Fe/Al molar ratios. The nitrogen adsorption-desorption isotherm is classified as H1 type. The hysteresis curves for samples FA1100-1 and FA1100-2 are presented in Figure 10c. Notably, the nitrogen adsorption-desorption curve for FA1100-2 is categorized as H2 type, while both materials exhibit stepped and uniform reaction pore diameters. H1 type hysteresis loops are indicative of mesoporous structures, akin to ink bottle pores, whereas H2 type isotherms suggest typical physical adsorption processes occurring on non-porous or microporous adsorbents [34]. It is widely recognized that an increase in adsorption volume corresponds to an expansion of pore space and an enhancement in adsorption capacity [35]. The data presented in Figure 10b,c indicate that the pore size distribution of the synthesized Fe_2_O_3_/γ-Al_2_O_3_ ranges from 5 to 50 nm, thereby providing further evidence that these materials are classified as mesoporous. Additionally, as detailed in Table 5 and Table 6, S_BET_ is a critical factor influencing equilibrium adsorption capacity, with materials exhibiting a larger S_BET_ demonstrating an enhanced equilibrium adsorption capacity.

### 3.2. The Process of Adsorption of Congo Red Utilizing the Synthesized Adsorbent

**Figure 11:**
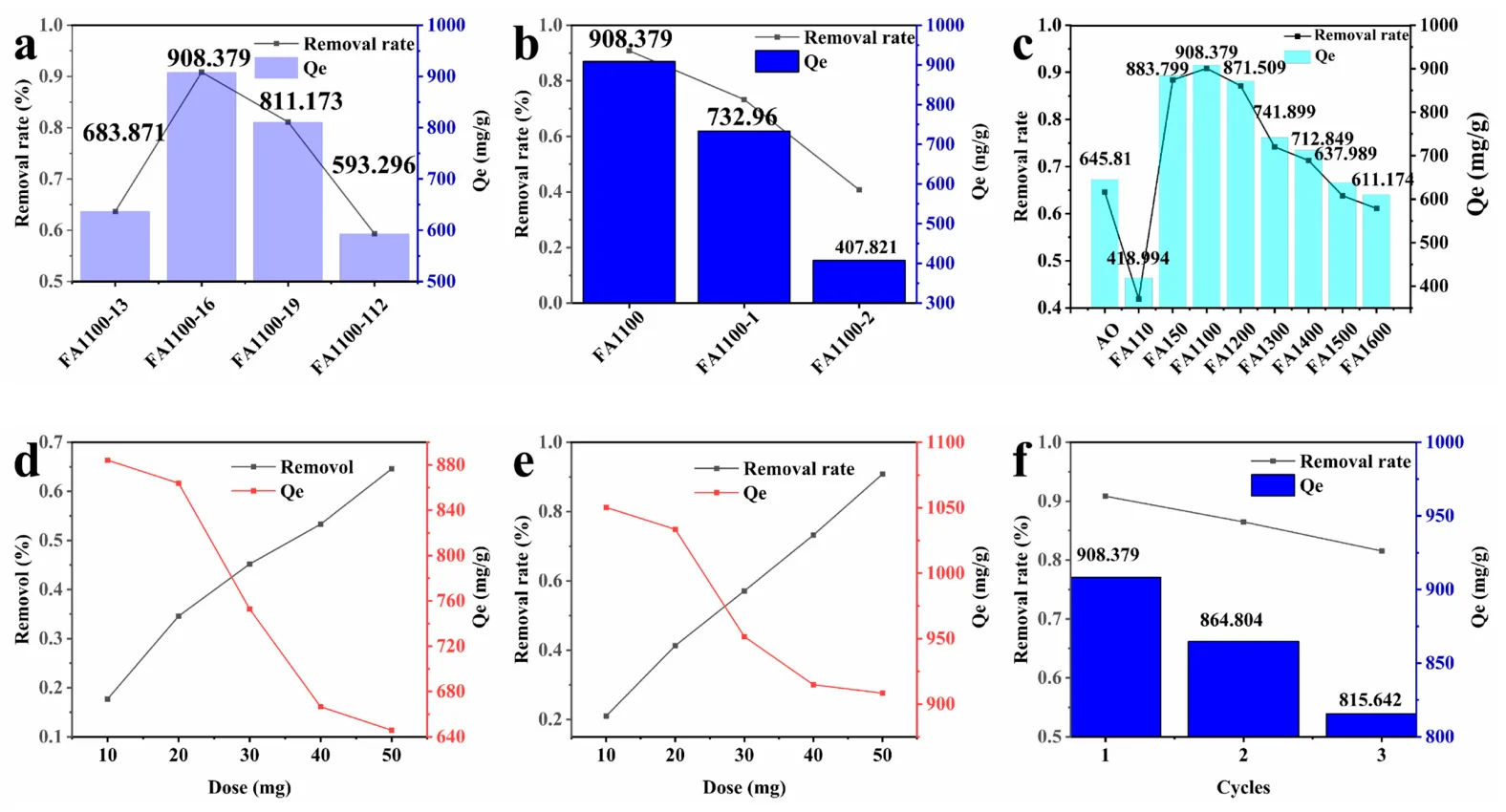
(**a**) Adsorption capacity and removal rate of FA1100 at different alcohol-to-water molar ratios; (**b**) Comparison of adsorption capacity using two iron sources and two aluminum sources; (**c**) Comparison of adsorption capacity at different Fe/Al molar ratios; (**d**,**e**) Effects on adsorption capacity of AO and FA1100, respectively; (**f**) Adsorption capacity of FA1100 after three regeneration cycles. (CR concentration: 1000 mg/L, volume: 50 mL).

The initial focus of this study was to investigate the impact of the molar ratio of n-hexanol to water on the synthesis of FA1100 composites. A series of FA1100 samples were synthesized at varying alcohol-to-water ratios, specifically ranging from 1:3 to 1:12, to assess their pore volume, pore size, specific surface area, and the subsequent implications for adsorption performance. A sample weighing 50 mg was immersed in a 1000 mg/L CR solution for a duration of 72 h, after which BET analysis was conducted on the sample. The findings of this research are presented in Table 7 of Figure 11a. The results indicate that at an alcohol-to-water ratio of 1:6, the adsorption capacity achieved peak values of 908.38 mg/g. S_BET_ was measured at 246.22 m^2^/g, with a pore volume of 0.56 cm^3^/g and a pore diameter of 6.65 nm. This suggests that the proportion of water to ROH within the system influences the dissociation of the OR group from the aluminum alkoxide, as well as the directional pore-forming properties of the OR group [36]. It is important to note that FA1100 corresponds to a molar ratio of Fe/Al of 1:100, and the designation 16 signifies a molar ratio of alcohol to water of 1:6. All subsequent experiments utilized composite materials synthesized at the alcohol-to-water ratio of 1:6. Consequently, FA1100-16 will be referred to simply as FA1100 in the remainder of this study.

To investigate the impact of iron and aluminum from various sources on the adsorption capacity of CR, samples FA1100, FA1100-2, and FA1100-1 were each introduced into a 1000 mg/L CR solution at a concentration of 50 mg. Following a stirring period of 72 h, *Q_e_* were determined to be 908.38, 732.96, and 407.82 mg/g, respectively. As illustrated in Figure 11b and detailed in Table 6, S_BET_ values for FA1100, FA1100-1, and FA1100-2 were recorded as 246.22 m^2^/g, 206.35 m^2^/g, and 173.09 m^2^/g, respectively. Notably, in comparison to FA1100, both the pore volume and specific surface area of FA1100-1 exhibited a reduction. This decrease may be attributed to the hydrolysis process that produces Al(OH)_3_OR, where R denotes –CH(CH_3_)_2_ and –(CH_2_)_5_CH_3_, respectively. The findings suggest that the pore-forming capacity of the –(CH_2_)_5_CH_3_ group is superior to that of the –CH(CH_3_)_2_ group [36]. In contrast to the heterogeneous reaction observed with FA1100, the hydrolysis of FA1100-2 is characterized as a homogeneous reaction. In this case, both aluminum n-hexanol and iron ethoxide undergo simultaneous hydrolysis at a consistent rate, resulting in larger pore sizes and volumes compared to FA1100, although the specific surface area is smaller.

Investigating the Fe_2_O_3_ modification level is essential for optimizing FA1100 synthesis and CR adsorption capacity. A series of Fe_2_O_3_/γ-Al_2_O_3_ composites with varying Fe/Al ratios was evaluated for CR adsorption (Figure 11c). Adsorption capacity increased with Fe content, peaking at an Fe/Al molar ratio of 1:100, beyond which it declined. Based on BET, EPR, and XPS analyses, this trend is governed by two opposing factors. First, Fe incorporation introduces Fe–O–Fe surface species, increasing specific surface area. Second, excessive Fe leads to atomic substitution of Al by Fe (due to Fe’s larger atomic radius), forming Fe–O–Al bonds that cause pore blockage, reducing pore volume, pore diameter, and surface area. While increased surface area and active sites enhance chemisorption, pore blockage imposes an upper limit on overall adsorption performance. Thus, the Fe/Al ratio of 1:100 was identified as optimal, and the corresponding composite was designated FA1100 (Figure 11c).

Adsorbent dosage significantly influences the overall cost of treatment equipment, making it imperative to evaluate its effect on CR adsorption efficiency. In this study, the adsorption of CR by FA1100 and AO was assessed at dosages ranging from 10 to 50 mg per 50 mL, with a fixed CR concentration of 1000 mg/L. As shown in Figure 11d,e, increasing the dosage to 50 mg/50 mL markedly enhanced CR adsorption, attributed to the greater availability of active surface sites, which promotes stronger adsorbent–solute interactions and provides more adsorption sites. Accordingly, a dosage of 50 mg/50 mL was selected as optimal for subsequent experiments.

The adsorption stability of FA1100 was evaluated through recovery experiments using 1000 mg/L CR solution (Figure 11f). After each adsorption cycle, the dye-adsorbent mixture was centrifuged at 8000 rpm for 10 min at ambient temperature. The adsorbent was regenerated in a muffle furnace following the established calcination protocol, and cyclic adsorption was performed twice under identical conditions. Results show that even after three cycles, the *Q_e_* of FA1100 for CR remains 815.64 mg/g, indicating sustained effective adsorption throughout the recycling process.

It should be noted that the regeneration experiment was performed for only two cycles due to time and resource constraints, and the method employed—calcination at elevated temperature—is energy-intensive and may not represent a practical reuse strategy for environmental remediation. While the stable adsorption capacity over the two cycles confirms the chemical robustness of the composite, extended cycling tests (at least five cycles) are required to more comprehensively assess its long-term reusability. Additionally, the calcination step removes adsorbed CR molecules through thermal decomposition but raises concerns regarding energy cost, potential structural changes (e.g., pore collapse or Fe species aggregation), and material stability over repeated cycles. We acknowledge that milder regeneration approaches (e.g., solvent desorption using NaOH or ethanol) were not explored in the present work, though such methods would be more economically and environmentally viable for practical applications. Therefore, while the present results demonstrate the regenerability of the material under ideal laboratory conditions, further studies are needed to develop and evaluate more energy-efficient and practically feasible regeneration protocols. We have noted this as a key direction for future research.

**Figure 12:**
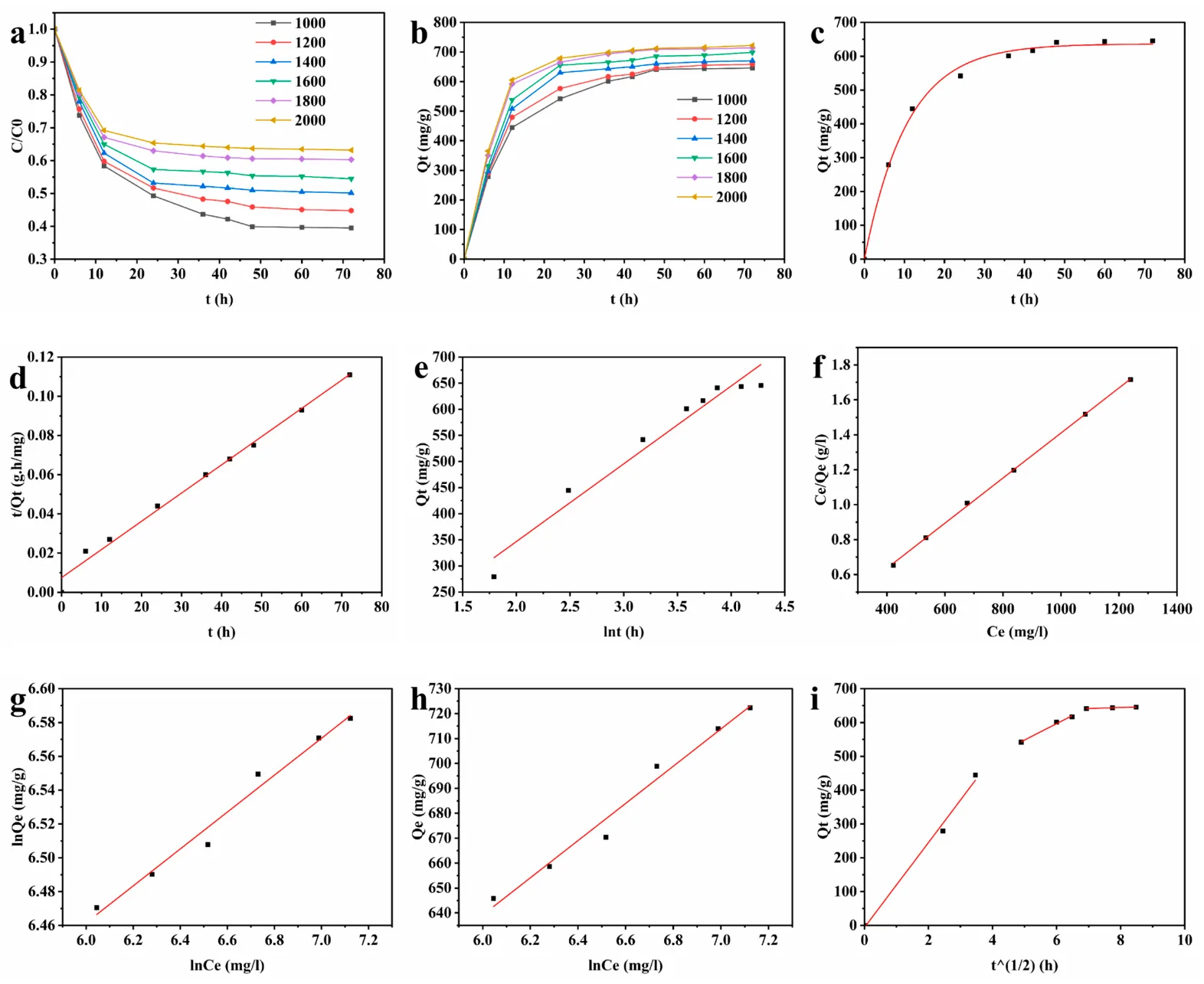
AO (**a**,**b**). The changes in relative concentration and adsorption capacity over time, (**c**) PFO, (**d**) PSO, (**e**) Elovich, (**f**) Langmuir, (**g**) Freundlich, (**h**) Temkin model, and (**i**) Weber–Morris intra-particle diffusion model.

**Figure 13:**
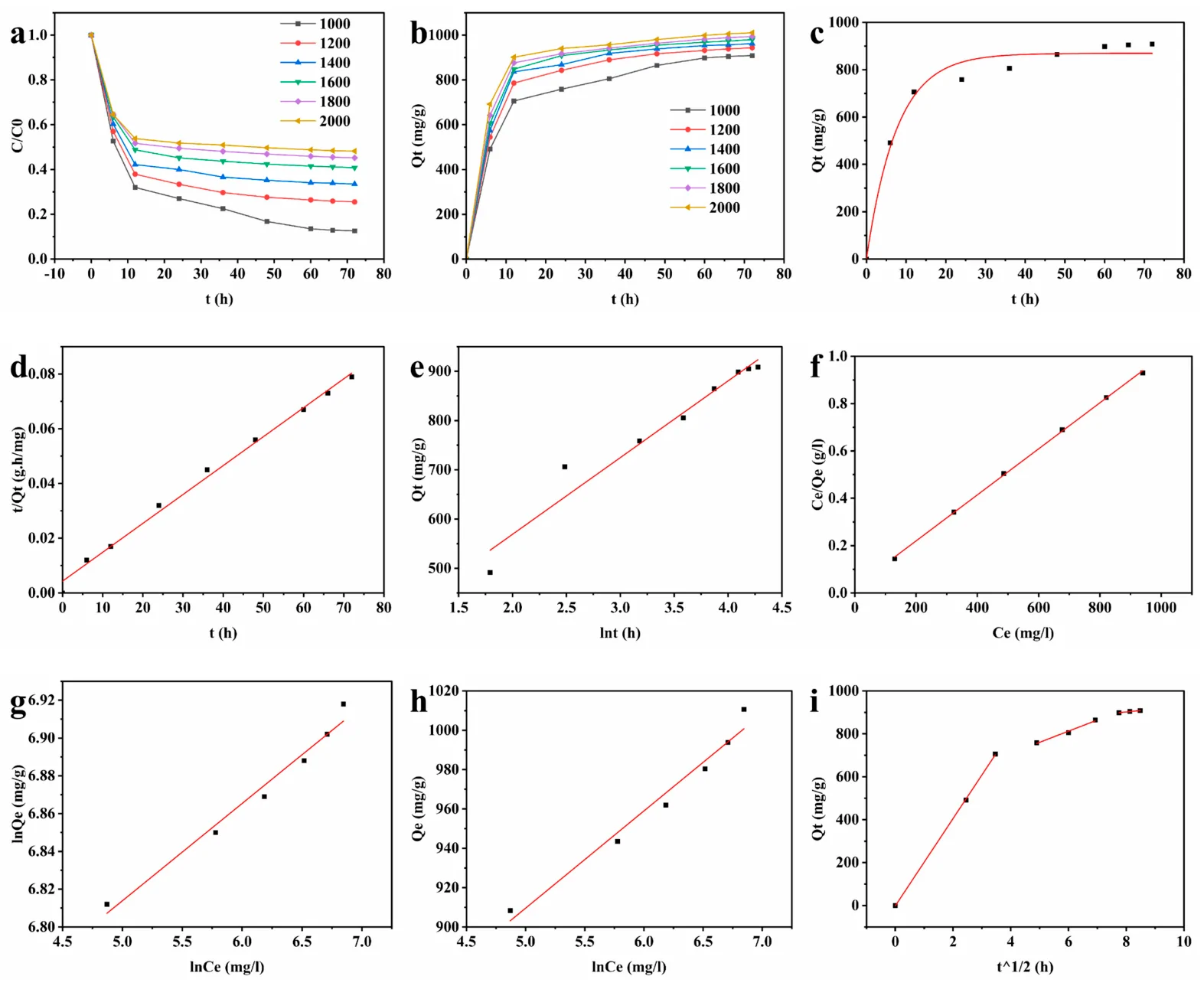
FA1100 (**a**,**b**). The changes in relative concentration and adsorption capacity over time, (**c**) PFO, (**d**) PSO, (**e**) Elovich, (**f**) Langmuir, (**g**) Freundlich, (**h**) Temkin model, and (**i**) Weber Morris intra-particle diffusion model.

To investigate adsorption pathways and kinetics, time-dependent experiments were conducted over 0–72 h for FA1100 and AO (Figure 12a,b and Figure 13a,b, Table 8). At an adsorbent dosage of 50 mg/50 mL and a CR concentration of 1000 mg/L, AO reached equilibrium within 48 h (*Q_t_* = 641.34 mg/g), while FA1100 required 60 h (*Q_t_* = 898.32 mg/g). Higher CR concentrations led to increased equilibrium capacity due to surface site saturation. Given intraparticle diffusion, the adsorption duration was set at 72 h. The smaller pore volume and size of FA1100 (Table 8) contribute to its longer equilibration time compared to AO. Three kinetic models—pseudo-first-order (PFO), pseudo-second-order (PSO), and Elovich—were employed to elucidate the adsorption mechanism and rate-controlling efficiency. The kinetic data for AO fit the PFO model well (R^2^ = 0.995), outperforming the PSO model. For FA1100, the PSO model provided a better fit (R^2^ = 0.993) than the PFO model, indicating chemisorption involvement. The optimal kinetic parameters are summarized in Table 8. The PFO rate constant *k*_1_ for AO is 0.0934 h^−1^, while the PSO rate constant *k*_1_ for FA1100 is 0.00026 g/(mg·h). The rapid CR adsorption by FA1100 is attributed to its abundant active sites, mesoporous structure, and high specific surface area, which enhance intraparticle fluidity.

To evaluate the effect of temperature on adsorption capacity, the Langmuir, Freundlich, and Temkin isotherm models were applied to analyze CR adsorption on AO and FA1100 at 25 °C (Figure 12f–h and Figure 13f–h; fitting parameters in Table 8). The Langmuir model provided superior fits for both adsorbents (R^2^ = 0.999 for AO, 0.999 for FA1100) compared to the Freundlich and Temkin models, indicating monolayer uniform adsorption. The Langmuir-derived *Q_m_* were 775.19 mg/g for AO and 1027.72 mg/g for FA1100, representing a 32.58% increase for FA1100. This capacity surpasses that of many previously reported adsorbents (Table 8). The mesoporous composite FA1100, synthesized via a straightforward in situ method, exhibits significant CR adsorption capacity, highlighting its promising potential for practical applications.

Meanwhile, the intra-particle diffusion model was employed to investigate the transport mechanism of CR within AO and FA1100. The findings are illustrated in Figure 12i and Figure 13i and summarized in Table 8. The R^2^ values obtained from the intra-particle diffusion model were 0.984, 0.979, 0.998, and 0.999, 0.973, 0.938, respectively, suggesting that the adsorption process of CR aligns with the intra-particle diffusion model. Furthermore, the calculated rate constants (k) were 125.73, 48.48, 2.88, 203.28, 52.02, 13.64, respectively. The rate constants suggest that the adsorption process of CR onto FA1100 and AO can be delineated into three distinct phases. The initial phase is characterized by the rapid adsorption of CR molecules onto the active surface sites of FA1100 and AO. The subsequent phase involves a reduction in the adsorption rate, which is attributed to a decline in the concentration of CR. The final phase encompasses the diffusion of CR from the surface into the internal pores, ultimately leading to the attainment of adsorption equilibrium.

**Figure 14:**
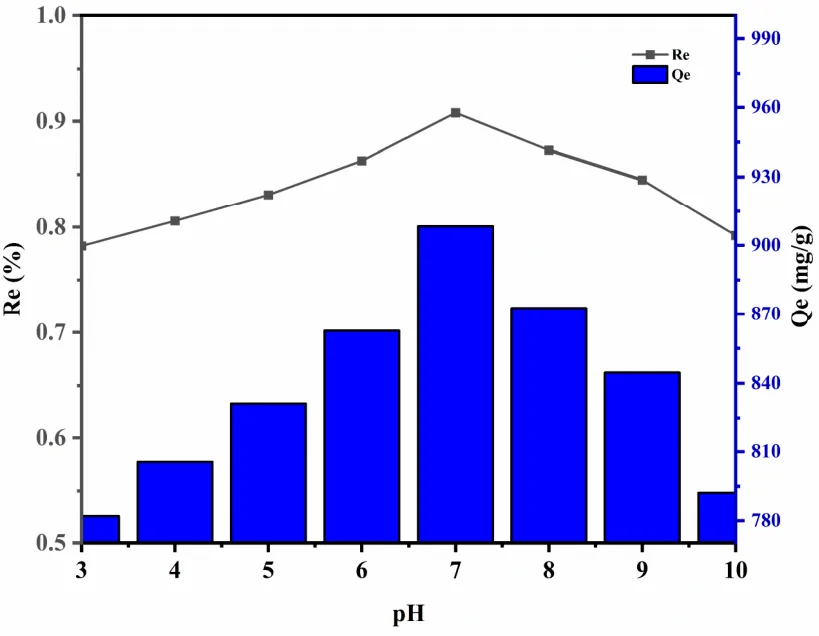
The influence of pH on the adsorption performance of FA1100.

To investigate the potential impact of electrostatic interactions on the adsorption characteristics of FA1100, an experimental setup was established in which 50 mg of FA1100 was added to solutions of CR with varying initial pH levels. The findings are illustrated in Figure 14. The adsorption efficiency remains relatively high under weakly acidic, neutral, and weakly alkaline conditions. Given that a neutral pH is representative of typical aquatic environments, the objective of utilizing FA1100 is to optimize the adsorption conditions to enhance its adsorption efficacy. Notably, the surface of FA1100 possesses a positive charge, which, in conjunction with the anionic nature of CR, suggests that at a pH of 7, a neutral aqueous environment facilitates electrostatic attraction, thereby improving the equilibrium adsorption capacity. It holds significant potential for practical applications.

Finally, the potential applications of Fe_2_O_3_/γ-Al_2_O_3_ across various fields were discussed, and a comparative analysis of the adsorption effects of γ-Al_2_O_3_ synthesized with Fe_2_O_3_/γ-Al_2_O_3_ as the carrier, alongside other adsorbents utilizing γ-Al_2_O_3_ as the carrier on CR, was conducted, as well as the adsorption performance of various other adsorbents on CR. The specifics of these findings are presented in Table 9.

### 3.3. Reaction Mechanism

**Figure 15:**
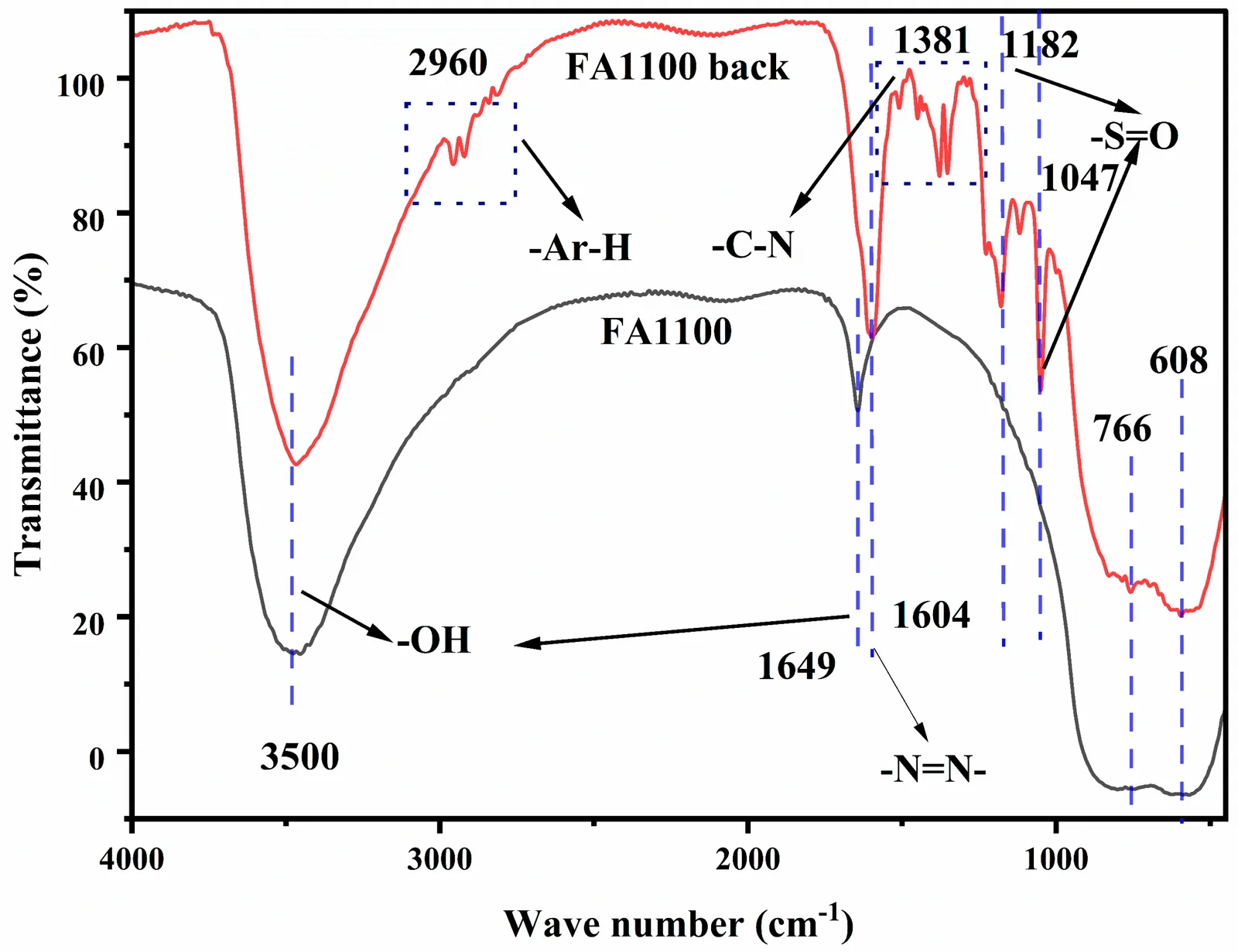
Comparison chart of FT-IR before and after CR adsorption by FA1100.

**Figure 16:**
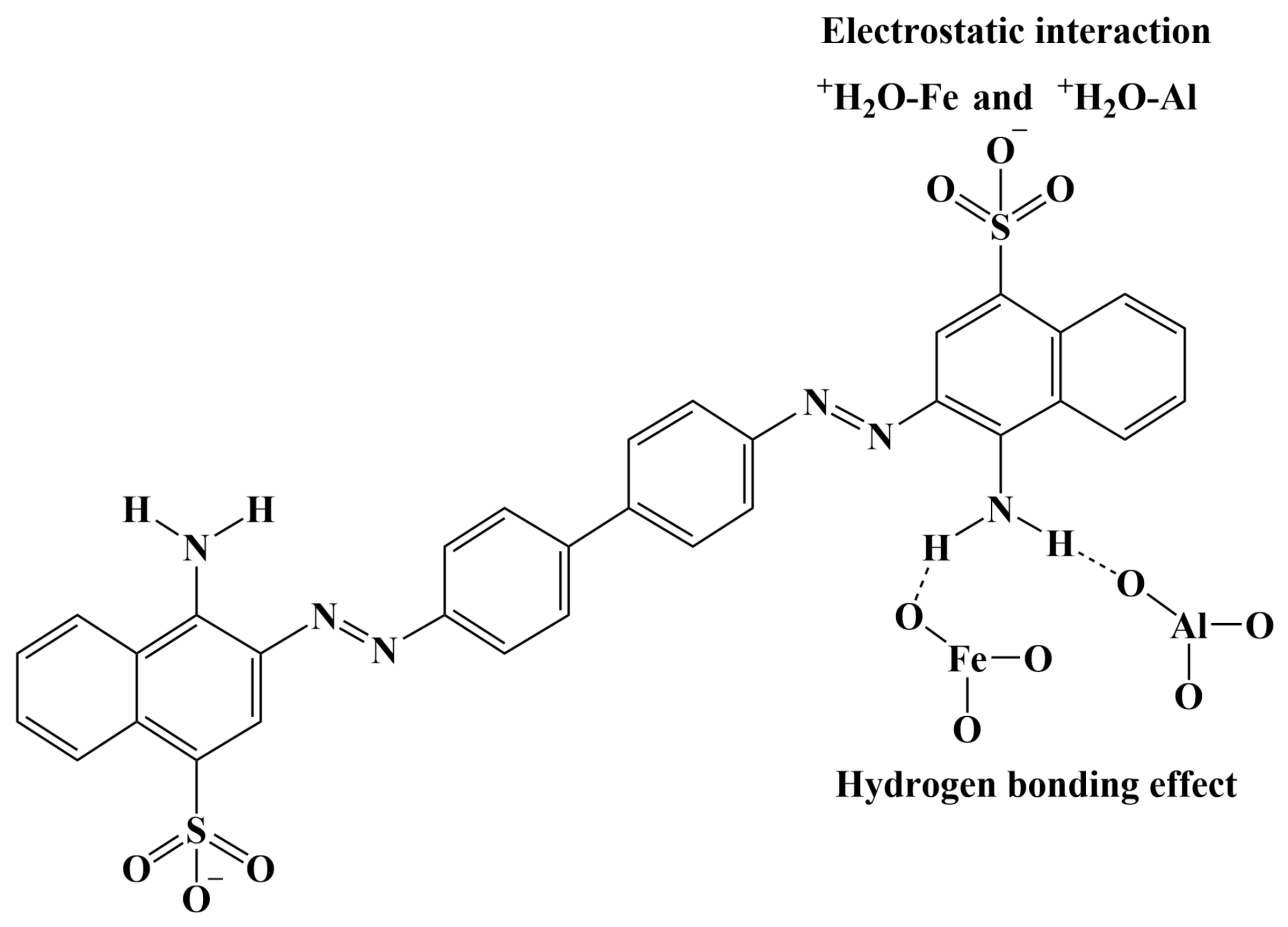
The mechanism of CR adsorption by FA1100.

In the investigation of adsorption kinetics, the adsorption data for AO and FA1100 were found to be better described by the pseudo-first-order and pseudo-second-order kinetic models, respectively. Notably, the DFT-calculated adsorption energies for CR on FA1100 (−1.58 eV) are significantly more negative than those on AO (−1.03 eV), indicating stronger binding interactions on the Fe_2_O_3_/γ-Al_2_O_3_ heterojunction. The relatively large magnitude of these adsorption energies, combined with the good fit to the pseudo-second-order model, provides supporting evidence for the involvement of chemisorption in the adsorption process, wherein surface interactions and site availability play a dominant role in determining the adsorption rate. This observation suggests that chemical adsorption is the predominant mechanism in the adsorption process. The phenomenon of chemical adsorption can be described in terms of Lewis acid-base interactions. Within Fe/Al nanocomposites, coordination occurs with water molecules present in the Congo red solution, resulting in the formation of Fe–OH_2_^+^ and Al–OH_2_^+^ species, which function as hard Lewis acid sites. Concurrently, the –NH_2_ groups on the Congo red molecules serve as hard Lewis base sites [48]. According to Pearson’s Hard-Soft Acid-Base (HSAB) theory, these entities exhibit a propensity to form robust bonds, resulting in the formation of ionic complexes. Furthermore, the adsorption isotherm data for both materials were reasonably described by the Langmuir model, suggesting that monolayer-type adsorption on energetically similar sites may be a significant feature of the adsorption process. However, given the mesoporous structure and Fe-modified, defect-containing nature of FA1100, the surface is likely heterogeneous. Therefore, while the Langmuir fit provides a useful description of the equilibrium data, it should not be interpreted as definitive proof of a perfectly uniform surface or an exclusive monolayer mechanism. The Freundlich model was also considered to account for potential adsorption on heterogeneous sites. Taken together, the isotherm analysis indicates that monolayer-type adsorption on the most favorable sites likely contributes significantly to the overall uptake, alongside other possible adsorption modes. Additionally, both materials were found to comply with the Weber Morris intra-particle diffusion model; however, the fitting curve did not intersect the origin, implying the presence of an alternative adsorption mechanism. As an anionic dye, CR possesses a negative charge. The findings presented in Figure 13 indicate that under strongly acidic conditions, H^+^ interact with the –SO_3_^2−^ groups on the surface of CR, thereby diminishing the interaction between FA1100 and –SO_3_^2−^ and subsequently reducing the adsorption capacity [49]. In contrast, under weakly acidic and neutral conditions, the positive charge on the surface of FA1100 and the negative charge of CR facilitate electrostatic attraction during the adsorption process. In alkaline environments, given that the zero charge point of Al_2_O_3_ is 9 and that of Fe_2_O_3_ is 7 [50], while the zero charge points of AO and FA1100 are 8.8 and 8.2 respectively, the negatively charged surface of FA1100 experiences electrostatic repulsion from CR, which leads to a decrease in equilibrium adsorption capacity. Simultaneously, the ZETA potential analyzer determined that both AO and FA1100 exhibited positive surface charges of 29.4 mV and 36.9 mV (in Table 10), respectively. These findings suggest that the incorporation of Fe_2_O_3_ has increased the positive charge of the composite material. Furthermore, this observation substantiates the presence and amplification of electrostatic interactions between FA1100 and CR throughout the adsorption process. FT-IR spectroscopy was performed on FA1100 both prior to and following CR adsorption, with the corresponding spectra presented in Figure 15. The absorption bands observed near 1047 cm^−1^ and 1182 cm^−1^ are attributed to the stretching vibrations of –S=O. A peak at approximately 1381 cm^−1^ corresponds to the –C–N functional group, while the band near 2960 cm^−1^ is assigned to –Ar–H stretching vibrations. –OH exhibits characteristic stretching and bending vibrations at 3500 cm^−1^ and 1649 cm^−1^, respectively. Additionally, the absorption band around 1604 cm^−1^ is associated with –N=N–, and the peaks near 766 cm^−1^ and 608 cm^−1^ are indicative of Al–O bonds. The bands at 3500 cm^−1^, 1649 cm^−1^, 1766 cm^−1^, and 608 cm^−1^ correspond to the characteristic structural features of γ-Al_2_O_3_ present in FA1100. Following CR adsorption, an enhancement in the -OH stretching vibration peak was observed, alongside a strengthening and shift of the bending vibration peak related to the -N=N- of CR. These spectral changes suggest the formation of interactions between hydroxyl groups and azo functionalities, indicating a combination or bonding between –OH and –N=N– groups during the adsorption process. This enhancement implies that FA1100 interacts with water to form Fe–OH^2+^/Al–OH^2+^ complexes, which subsequently engage with the -NH_2_ groups of CR to establish hydrogen bonds. Consequently, this evidence supports the conclusion that the chemical mechanism underlying the adsorption of CR by FA1100 is primarily through hydrogen bond formation [51].The mechanism diagram is shown in Figure 16.

### 3.4. DFT Calculation

All spin-polarized density functional theory (DFT) calculations were performed using the Vienna Ab initio Simulation Package (VASP). The projector augmented wave (PAW) method was used to describe the ion–electron interactions. The Perdew–Burke–Ernzerhof (PBE) functional within the generalized gradient approximation (GGA) was employed for the exchange-correlation energy, with the Grimme D3 dispersion correction included to account for van der Waals interactions. The plane-wave cutoff energy was set to 500 eV. A Gamma-centered k-point mesh with a reciprocal-space resolution of 0.04 Å^−1^ was used for Brillouin zone sampling. The electronic energy convergence criterion was set to 1 × 10^−5^ eV, and geometry optimization was performed until the force on each atom was less than 0.03 eV/Å. Spin polarization was considered for all Fe-containing models, with initial magnetic moments assigned to Fe atoms and allowed to relax during structural optimization. The DFT+U method was applied with an effective U value of 4.0 eV for Fe 3d electrons. The anionic form of Congo red (CR^2−^) was used in the calculations to reflect its charge state under the experimental conditions. Solvent effects and counterions were not explicitly included; therefore, the DFT results presented here should be regarded as qualitative, providing insights into relative trends rather than absolute predictions.

**Figure 17:**
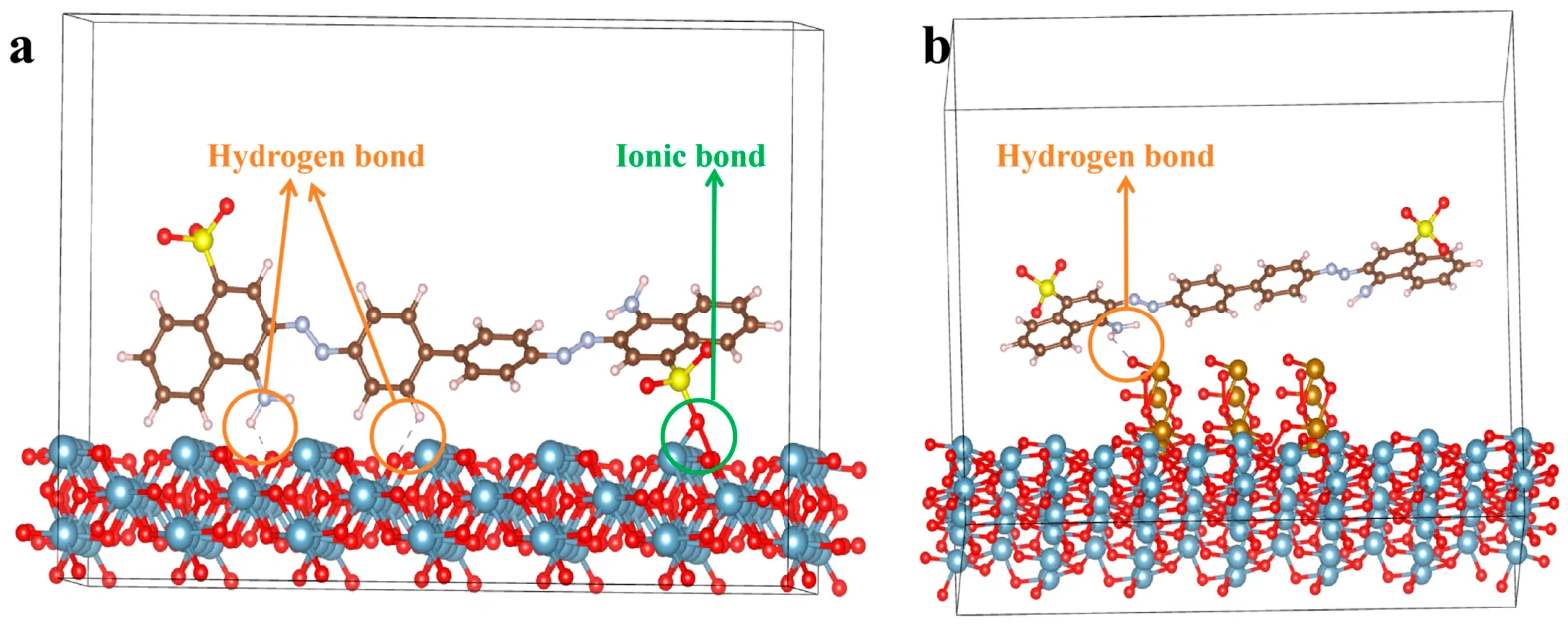
Model diagram of (**a**) AO and (**b**) FA1100 adsorbing Congo Red.

To elucidate the adsorption mechanism of AO and FA1100 toward Congo red, density functional theory (DFT) calculations were performed. In these computational models, FA1100 was represented as a heterojunction structure, wherein the substrate consisted of γ-Al_2_O_3_ constructed along the (1, 0, 0) lattice plane, and the cluster component comprised Fe_2_O_3_. A detailed schematic of the structural configuration is presented in Figure 17. The computational results revealed that the adsorption energies of Congo red on AO and FA1100 were −1.03 eV and −1.58 eV, respectively, while the interface energy of the heterojunction was calculated to be 0.08 eV/Å^2^. These findings suggest that FA1100 exhibits a stronger adsorption affinity for Congo red compared to AO. Additionally, Fe_2_O_3_ was found to form a thermodynamically weakly stable interface with γ-Al_2_O_3_. The relatively low interface energy facilitates the formation of a stable heterojunction, which in turn contributes to the preservation of the weak magnetic properties imparted by Fe_2_O_3_.

## 4. Discussion

In the present investigation, a mesoporous Fe_2_O_3_/γ-Al_2_O_3_ nanocomposite was synthesized successfully employing a straightforward sol-gel, one-pot approach. This nanocomposite exhibited a markedly enhanced adsorption capacity for Congo red dye (1027.72 mg/g) relative to pristine γ-Al_2_O_3_ (775.19 mg/g), which is attributed to its increased specific surface area (246.22 m^2^/g), mesoporous architecture, and the presence of oxygen vacancies alongside Fe–O–Al bond formation, as evidenced by XPS and EPR analyses. Kinetic evaluation indicated that the adsorption of Congo red onto the FA1100 sample conforms to a pseudo-second-order kinetic model (R^2^ = 0.993), suggesting that chemisorption predominates the adsorption mechanism. Furthermore, equilibrium data were best described by the Langmuir isotherm model (R^2^ > 0.999), implying monolayer adsorption on a homogeneous surface. The Weber–Morris intraparticle diffusion model delineated a triphasic adsorption process encompassing an initial rapid surface adsorption phase, followed by gradual intraparticle diffusion, and culminating in equilibrium. Experiments varying solution pH highlighted the role of electrostatic interactions in the adsorption process. Regeneration studies demonstrated that FA1100 maintained approximately 79.4% of its original adsorption capacity after three successive cycles, indicating notable reusability. Complementary DFT calculations substantiated the experimental observations by revealing enhanced binding affinities attributable to Fe–O–Al linkages and oxygen vacancies. Compared to conventional adsorbents such as activated carbon, which are often limited by high costs and challenging regeneration procedures, FA1100 presents a more sustainable and economically viable alternative. It is important to note that the adsorption experiments in this study were conducted under simplified laboratory conditions (high CR concentrations, long contact times, and simple aqueous systems without competing ions or natural organic matter). Therefore, while the Fe_2_O_3_/γ-Al_2_O_3_ composite demonstrates considerable potential as an adsorbent for anionic dyes, further research is warranted to evaluate its performance in more realistic water matrices, assess its long-term stability and reusability under field conditions, and optimize the synthesis method for scalability. In summary, this work provides a comprehensive mechanistic understanding of the adsorption process and establishes the Fe_2_O_3_/γ-Al_2_O_3_ composite as a promising candidate that merits further investigation for practical environmental applications.

The exceptionally high CR adsorption capacity of FA1100 (1027.72 mg/g) arises from the synergistic combination of four key mechanisms. First, pore filling contributes to the overall uptake, as the mesoporous structure (S_BET_ = 246.22 m^2^/g, pore volume = 0.558 cm^3^/g, average pore diameter = 6.65 nm) provides abundant spaces for CR molecules to diffuse into and become physically entrapped within the pore channels. Second, electrostatic attraction plays a supportive role; zeta potential measurements revealed positive surface charges of 29.4 mV and 36.9 mV for AO and FA1100 at neutral pH, with pHpzc (point of zero charge) values of approximately 8.8 and 8.2, respectively, favoring attraction with the negatively charged CR molecules under weakly acidic to neutral conditions. Third, hydrogen bonding serves as the primary mechanism, as the abundant hydroxyl groups on the FA1100 surface form strong hydrogen bonds with the sulfonic acid (–SO_3_), amino (–NH_2_), and azo (–N=N–) groups of CR, supported by the DFT-calculated adsorption energy of –1.58 eV. Fourth, Fe–O–Al active sites provide additional chemical interactions; the highly dispersed Fe species (likely as FeOₓ, isolated Fe(III) ions, or Fe–O–Al surface species) introduce active sites that enhance adsorption affinity through surface complexation and Lewis acid–base interactions, with the heterojunction interface (interface energy = 0.08 eV/Å^2^) facilitating charge transfer and synergistic electronic effects. The combination of these physical and chemical forces ensures that multiple adsorption pathways operate simultaneously, collectively accounting for the superior adsorption capacity observed in this work.

To further elucidate the adsorption mechanism at the molecular level, DFT calculations were performed on both AO (γ-Al_2_O_3_) and FA1100 (Fe_2_O_3_/γ-Al_2_O_3_ heterojunction) models. The computational results revealed that the adsorption energy of CR on FA1100 (−1.58 eV) is significantly more negative than that on AO (−1.03 eV), indicating that the incorporation of Fe_2_O_3_ substantially enhances the adsorption affinity of the composite material. This enhancement is attributed to the synergistic electronic interactions at the heterojunction interface, where the Fe_2_O_3_ cluster and the γ-Al_2_O_3_ substrate jointly contribute to the binding of CR molecules. The interface energy of the heterojunction was calculated to be 0.08 eV/Å^2^, suggesting a thermodynamically stable interface that facilitates efficient charge transfer and stabilizes the composite structure.

Importantly, the DFT findings are fully consistent with the experimental results. The more negative adsorption energy calculated for FA1100 correlates well with its experimentally determined higher adsorption capacity (1027.72 mg/g) compared to pristine AO, confirming that the heterojunction structure plays a pivotal role in enhancing CR uptake. The chemisorption-dominated mechanism suggested by the pseudo-second-order kinetic model and Langmuir isotherm is further supported by the relatively strong adsorption energies derived from DFT calculations. Moreover, the stable heterojunction interface, as indicated by the low interface energy, not only contributes to the adsorption performance but also helps to preserve the magnetic properties imparted by Fe_2_O_3_, as confirmed by the VSM measurements. Collectively, the combined DFT and experimental analyses provide a comprehensive mechanistic understanding, wherein the high CR adsorption capacity of FA1100 arises from the synergistic effects of pore filling, electrostatic attraction, hydrogen bonding, surface complexation, and the enhanced electronic interactions at the heterojunction interface.

The adsorption behavior of CR on FA1100 is governed primarily by hydrogen bonding, with electrostatic attraction playing a secondary role. The zeta potential measurements reveal that AO and FA1100 exhibit positive surface charges of 29.4 mV and 36.9 mV, respectively, at neutral pH, while the pHpzc of AO and FA1100 are approximately 8.8 and 8.2. These surface charge properties directly influence the electrostatic interactions with the anionic CR molecules. At pH values below the pHpzc, the positively charged adsorbent surface favors electrostatic attraction with CR, contributing to the high adsorption capacity observed at neutral pH. Conversely, when the pH exceeds the pHpzc, the surface becomes negatively charged, leading to electrostatic repulsion and reduced uptake-a trend consistent with the pH-dependent adsorption experiments. However, the fact that substantial adsorption is maintained even under alkaline conditions suggests that electrostatic attraction is not the sole or dominant driving force. Instead, the abundant hydroxyl groups on the FA1100 surface and the multiple functional groups of CR (–SO_3_, –NH_2_, –N=N–) facilitate strong hydrogen bonding interactions, which provide a more pH-independent contribution. The DFT-calculated adsorption energy of −1.58 eV further supports the strength of these interactions. Thus, we propose that hydrogen bonding is the primary mechanism, while electrostatic attraction, modulated by zeta potential and pHpzc, plays a supportive and pH-dependent role in the overall adsorption process.

The active sites responsible for CR adsorption on the Fe_2_O_3_/γ-Al_2_O_3_ composite can be identified by combining experimental characterization with DFT calculations. The DFT results reveal that CR molecules preferentially adsorb on the Fe sites of the Fe_2_O_3_ cluster within the heterojunction, with a calculated adsorption energy of −1.58 eV—significantly stronger than that on pristine γ-Al_2_O_3_ (−1.03 eV). This indicates that the Fe species introduce primary binding sites through surface complexation between the Fe cations and the sulfonic acid groups of CR molecules. In addition, the abundant surface hydroxyl groups on both γ-Al_2_O_3_ and Fe_2_O_3_ serve as secondary active sites, facilitating hydrogen bonding interactions with the amino groups and sulfonic acid groups of CR. Furthermore, the heterojunction interface between Fe_2_O_3_ and γ-Al_2_O_3_, with a calculated interface energy of 0.08 eV/Å^2^, contributes synergistic electronic effects that enhance the surface charge density and create additional favorable adsorption sites, as supported by the charge density difference analysis. The combination of these active sites—Fe sites, hydroxyl groups, and the heterojunction interface—collectively accounts for the superior CR adsorption performance of the Fe_2_O_3_/γ-Al_2_O_3_ composite.

A direct correlation between experimental and theoretical findings can be established to validate the proposed adsorption mechanism. First, the experimentally determined adsorption capacity of FA1100 (1027.72 mg/g) is significantly higher than that of pristine AO, which is fully consistent with the DFT-calculated adsorption energies (−1.58 eV for FA1100 vs. −1.03 eV for AO). This quantitative agreement confirms that the enhanced molecular-level binding affinity on the Fe_2_O_3_/γ-Al_2_O_3_ heterojunction directly translates to the superior macroscopic adsorption performance. Second, the pseudo-second-order kinetic model and Langmuir isotherm suggest a chemisorption-dominated monolayer adsorption process, which is supported by the relatively strong adsorption energies derived from DFT calculations, indicating energetically favorable chemical interactions. Third, the pH-dependent adsorption experiments show maximum uptake at neutral pH, which correlates well with the DFT-derived charge density distribution revealing favorable electrostatic interactions between the negatively charged CR molecules and the positively charged surface sites under neutral conditions. Fourth, the DFT-calculated interface energy of 0.08 eV/Å^2^ indicates a stable Fe_2_O_3_/γ-Al_2_O_3_ heterojunction, which is corroborated by experimental characterization (XRD, XPS, and TEM) confirming well-defined interfaces, and by VSM measurements showing preserved magnetic properties. The good agreement between experimental and theoretical results thus provides a robust validation of the proposed adsorption mechanism.

We acknowledge that the DFT model presented herein has certain limitations regarding its representation of aqueous adsorption conditions. Congo red is a large, charged anionic dye, and its adsorption behavior in water is influenced by multiple factors, including protonation state, the presence of counterions, hydration effects, and surface hydroxylation of the oxide adsorbent. Our current DFT model employs a simplified heterojunction slab geometry in a vacuum environment, without explicitly including solvent molecules, counterions, or surface hydration layers. As such, it does not fully capture the complexity of the aqueous adsorption environment. However, the primary purpose of these calculations is to provide qualitative insights into the relative adsorption affinities of CR on AO and FA1100, and to help interpret the experimental observations at the electronic level. The calculated trends—particularly the more negative adsorption energy for FA1100 compared to AO—are consistent with the experimental findings and support the conclusion that the Fe_2_O_3_/γ-Al_2_O_3_ heterojunction offers enhanced binding interactions. Nevertheless, we emphasize that the DFT results should be interpreted as complementary to, rather than independent evidence for, the experimental observations, and future work employing explicit solvation models (e.g., DFT with implicit solvation or ab initio molecular dynamics) would be valuable for a more realistic description of the aqueous adsorption process.

Furthermore, the adsorption performance in real wastewater matrices—which may contain competing ions and natural organic matter—remains to be evaluated in future studies.

## 5. Conclusions

In this study, mesoporous Fe_2_O_3_/γ-Al_2_O_3_ nanocomposites were successfully synthesized via sol–gel and one-pot methods using aluminum n-hexoxide and ferric chloride hexahydrate as precursors. The optimized composite exhibited a maximum CR adsorption capacity of 1027.72 mg/g, representing a 32.58% enhancement over pristine γ-Al_2_O_3_. BET analysis revealed a specific surface area of 246.22 m^2^/g, a pore volume of 0.558 cm^3^/g, and an average pore diameter of 6.65 nm. Adsorption kinetics followed the pseudo-second-order model, and the isotherm data were well fitted by the Langmuir model, indicating a chemisorption-dominated monolayer adsorption process. The Weber–Morris intraparticle diffusion model revealed a three-stage adsorption process, and the non-zero intercept suggested the involvement of additional boundary layer effects. pH-dependent adsorption behavior confirmed the presence of electrostatic interactions, while DFT calculations further demonstrated that the adsorption of CR is governed by the combined effects of chemical and physical forces.

The main advantages of this work include the use of an inexpensive and environmentally benign inorganic iron source (ferric chloride hexahydrate) instead of costly organic precursors, the systematic optimization of multiple synthesis parameters (alcohol-to-water ratio, aging time, and temperature), and the comprehensive mechanistic analysis integrating experimental and computational approaches. These contributions provide both fundamental insights into the structure–property–performance relationships of mixed-metal-oxide adsorbents and practical guidance for the design of cost-effective materials for anionic dye removal.

However, several limitations should be acknowledged. First, the adsorption experiments were conducted only at laboratory scale using synthetic CR solutions, without testing on real industrial wastewater matrices that may contain competing ions and organic matter. Second, the long-term stability and reusability of the composite were not systematically evaluated in this study.

Despite these limitations, the findings underscore the promise of the Fe_2_O_3_/γ-Al_2_O_3_ composite as a simple, cost-effective, and eco-friendly adsorbent for anionic dye removal. The mechanistic insights also contribute broadly to oxide-based adsorption systems. To contextualize its performance, Table 11 compares the specific surface area and maximum adsorption capacity of the synthesized composite with those of various Fe- and Al-based composite oxide counterparts. Evidently, the Fe_2_O_3_/γ-Al_2_O_3_ composite exhibits superior values in both metrics, a dual benefit that not only reflects greater site availability from the enlarged surface area but also suggests enhanced intrinsic affinity toward Congo red, likely attributable to the synergistic Fe–Al interplay within the oxide matrix. This comparison substantiates the competitive advantage of the present material and supports its viability as an efficient, low-cost alternative for anionic dye remediation.

For future prospects, we intend to extend this research in several directions: (1) evaluate the adsorption performance in real industrial wastewater to assess practical applicability; (2) systematically investigate the reusability and regeneration of the spent adsorbent; (3) explore the scalability of the synthesis method for potential industrial production; and (4) extend the present synthesis strategy to other mixed-metal-oxide systems (e.g., Fe_2_O_3_/TiO_2_, Fe_2_O_3_/CeO_2_) for the removal of various organic pollutants.

## Figures and Tables

**Table 1:** The formula utilizes the regression equation pertaining to the adsorption of Congo red.

No.	Equations	The Designation of the Formula.	The Significance of Parameters.
1	$Q_{e}=\frac{(C_{0}-C_{t})V}{m}$		The adsorption performance of the as-prepared adsorbents was evaluated using the following parameters. In the batch adsorption experiments, *C*_0_ and *C_e_* (mg/L) denote the initial and equilibrium CR concentrations in solution, respectively; *V* (L) is the solution volume; *m* (mg) is the adsorbent mass; *Q_t_* and *Q_e_* (mg/g) represent the CR uptake at time *t* and at equilibrium, respectively; and *R_e_* is the removal efficiency at equilibrium. For kinetic analysis, *k*_1_ (h^−1^) and *k*_2_ (g·mg^−1^·h^−1^) are the rate constants of pseudo-first-order and pseudo-second-order models, while α (g·mg^−1^·h^−1^) and β (g·mg^−1^) are the initial adsorption rate and desorption constant in the Elovich model. The intraparticle diffusion model is characterized by *k_w_* (mg·g^−1^·h^−0.5^) as the diffusion rate constant and *b* as the boundary layer intercept. For isotherm modeling, *Q_m_* (mg/g) is the monolayer adsorption capacity, *b* (L/mg) is the Langmuir constant related to the free adsorption energy.
2	$R_{e}=\frac{C_{0}-C_{e}}{C_{0}}\times 100\%$		
3	$Q_{t}=Q_{e}(1-e^{-k_{1}t})$	Pseudo-First- Order kinetics	
4	$\frac{t}{Q_{t}}=\frac{1}{k_{2}{Q_{e}}^{2}}+\frac{t}{Q_{e}}$	Pseudo-Second- Order kinetics	
5	$Q_{t}=\frac{1}{\beta }\ln(\alpha \beta )+\frac{1}{\beta }\ln t$	Elovich kinetics	
6	$\frac{C_{e}}{Q_{e}}=\frac{1}{K_{L}Q_{m}}+\frac{C_{e}}{Q_{m}}$	Langmiur model	
7	$\ln Q_{e}=\ln K_{F}+\frac{1}{n}\ln C_{e}$	Freundich model	
8	$Q_{e}=B\ln(A)+B\ln(C_{e})$	Temkin model	
9	$Q_{t}=k_{w}t^{0.5}+b$	Weber Morris intra-particle diffusion equation	

**Table 2:** The relevant parameters of XPS for FA110.

	BE [eV]	Atomic Conc. [%]
Fe 2p_3/2_	712.2	0.69
Al 2p	74.10	7.56
O 1s	531.3	12.33

**Table 3:** The EDS element content of FA1100 and AO.

Element	FA1100			AO		
	Apparent Concentration	wt%	Atomic Percentage %	Apparent Concentration	wt%	Atomic Percentage %
O	27.02	49.63	62.72	22.97	48.11	61
Al	44.69	49.16	36.84	43.16	51.89	39
Fe	1.08	1.2	0.43	0	0	0
Total	**-**	100	100	**-**	100	100

**Table 4:** The relevant parameters of ICP-OES for FA1100.

Sample ID	Line	Conc. (ppm)
S1-Fe Al—1	Fe 240.49	1.64
S1-Fe Al—1	Al 308.22	66.25
S1-Fe Al—2	Fe 240.49	1.61
S1-Fe Al—2	Al 308.22	66.18
S1-Fe Al—3	Fe 240.49	1.63
S1-Fe Al—3	Al 308.22	66.26

**Table 5:** S_BET_, Pore Volume and Pore Size with different molar ratios of Fe and Al.

Sample	S_BET_ (m^2^/g)	Pore Volume (cm^3^/g)	Pore Size (nm)
AO	202.27	1.44	23.48
FA1600	165.88	1.06	18.64
FA1500	177.37	1.10	18.53
FA1400	194.00	0.80	12.69
FA1300	195.00	0.56	8.32
FA1200	231.86	0.73	9.02
FA1100	246.22	0.56	6.65
FA150	231.28	0.59	7.23
FA110	166.22	0.29	4.94

The apertures mentioned throughout the text are all the average apertures.

**Table 6:** S_BET_, Pore Volume and Pore Size with different molar ratios of FA1100 were synthesized by two aluminum sources and two iron sources.

Sample	S_BET_ (m^2^/g)	Pore Volume (cm^3^/g)	Pore Size (nm)
FA1100	246.22	0.56	6.65
FA1100-1	206.35	0.49	6.82
FA1100-2	173.09	0.89	17.26

**Table 7:** S_BET_, Pore Volume and Pore Size of FA1100 with different alcohol-water ratios.

Sample	S_BET_ (m^2^/g)	Pore Volume (cm^3^/g)	Pore Size (nm)
FA1100-13	202.64	0.652	9.11
FA1100-16	246.22	0.56	6.65
FA1100-19	230.28	0.44	5.37
FA1100-112	196.84	0.42	6.03

**Table 8:** The relevant parameters of the pseudo-first-order model, pseudo-order stage model, Elovich model, as well as the Langmuir isothermal model, Freundlich isothermal model, Temkin isothermal model and intra-particle diffusion model of AO and FA1100. All data presented are from representative single measurements conducted under strictly controlled conditions. The observed trends were reproducible across multiple independent experiments.

Sample	Pseudo-first-order model			Pseudo-second-order model			Elovich model		
	*Q_e_*	*k*_1_	R^2^	*Q_e_*	*k*_2_	R^2^	α	β	R^2^
	mg g^−1^	h^−1^		mg g^−1^	g (mg h)^−1^		mg (g h)^−1^	g mg^−1^	
AO	636.59	0.0934	0.995	694.44	0.00040	0.990	206.85	0.0067	0.951
FA1100	869.18	0.1312	0.979	943.40	0.00026	0.993	815.06	0.0064	0.949
	Langmuir isothermal			Freundlich isothermal			Temkin isothermal		
	*Q_m_*	*K_L_*	R^2^	*K_F_*	n	R^2^	*A*	*B*	R^2^
	mg g^−1^	L mg^−1^					L mg^−1^		
AO	775.19	0.0107	0.999	332.44	9.1592	0.977	12.9538	74.657	0.975
FA1100	1027.72	0.0384	0.999	703.68	19.4137	0.971	662,437.07	49.424	0.964
	Weber Morris intra-particle diffusion model								
	*k*_3_	*b*_3_	R^2^	*k*_4_	*b*_4_	R^2^	*k*_5_	*b*_5_	R^2^
	mg (g h)^−1^			mg (g h)^−1^			mg (g h)^−1^		
AO	125.733	−6.4790	0.985	48.4833	305.9735	0.979	2.8678	621.44	0.998
FA1100	203.28	−1.407	1.000	52.0157	500.5882	0.973	13.6386	793.19	0.938

**Table 9:** The adsorption capacity of FA1100 for the removal of CR was assessed in comparison to recently documented adsorbents.

Adsorbent	Experimental Conditions				Adsorption Capacity *Q_m_* (mg/g)	References
	S_BET_ (m^2^/g)	Pore Volume (cm^3^/g)	Pore Size (nm)	Dose (mg)		
γ-Al_2_O_3_ (AO)	202.27	1.44	23.48	50	775.19	This work
γ-Fe_2_O_3_/γ-Al_2_O_3_ (FA1100)	246.22	0.56	6.65	50	1027.72	This work
Cr_2_O_3_/Al_2_O_3_ (60%)	66.9	0.143	3.74	None	54.61	[37]
Al_2_O_3_@ZnO	105	0.48	18.4	10	714	[38]
MAS	16.33	0.034	7.01	300	10.41	[39]
MWCNTs/LDHs	124.97	0.60	3.81	15	595.8	[40]
MIL-53(Fe)/chitosan	15.8	None	26.3	16	590.8	[41]
MXene/carbon foam	11.28	0.024	79.79	None	647.75	[42]
Co-MOF	9.28	0.38	4.19	10	976.92	[43]
NOBC	618.44	0.27	7.28	120	609.8	[44]
MOP	46.79	0.02	16.67	300	13.59	[45]
NH_2_(GL)-YSA@SZ4_10_	6.37	0.01	12.28	15	298.74	[46]
FA25(500)	210.93	None	11.48	None	941.54	[47]

**Table 10:** Zeta potential of AO and FA1100 at different pH values.

Sample	4	5	6	7	8	9	10
AO	43.5	36.9	28.8	18.2	10.1	−5.9	−19.8
FA1100	38.4	29.4	20.6	14.5	6.6	−8.34	−21.9

**Table 11:** The Comparison of the adsorption of CR by Fe-based and Al-based materials.

Adsorbent	Experimental Conditions				Adsorption Capacity *Q_m_* (mg/g)	References
	S_BET_ (m^2^/g)	Pore Volume (cm^3^/g)	Pore Size (nm)	Dose (mg)		
γ-Al_2_O_3_ (AO)	202.27	1.44	23.48	50	775.19	This work
γ-Fe_2_O_3_/γ-Al_2_O_3_ (FA1100)	246.22	0.56	6.65	50	1027.72	This work
PDFe/Al	37.58	0.062	16.47	1000	411	[52]
Cu/Fe/Al MMOs	132.01	0.27	1.43	1 mg/mL	111.4	[53]
Fe/MOF-5@CTS	516.54	None	2.40	None	219.78	[54]
ZnAl-MHA	38.39	0.18	15.23	None	178.57	[55]
Fe_3_O_4_@bacteria	26.40	None	11.84	10	320.20	[56]

## Data Availability

The raw data supporting the conclusions of this article will be made available by the authors on request.
